# Filamentous fungal carbon catabolite repression supports metabolic plasticity and stress responses essential for disease progression

**DOI:** 10.1371/journal.ppat.1006340

**Published:** 2017-04-19

**Authors:** Sarah R. Beattie, Kenneth M. K. Mark, Arsa Thammahong, Laure Nicolas Annick Ries, Sourabh Dhingra, Alayna K. Caffrey-Carr, Chao Cheng, Candice C. Black, Paul Bowyer, Michael J. Bromley, Joshua J. Obar, Gustavo H. Goldman, Robert A. Cramer

**Affiliations:** 1 Department of Microbiology and Immunology, Geisel School of Medicine at Dartmouth, Hanover, New Hampshire, United States of America; 2 Department of Molecular and Systems Biology, Geisel School of Medicine at Dartmouth, Hanover, New Hampshire, United States of America; 3 Faculdade de Ciências Farmacêuticas de Ribeirão Preto, Universidade de São Paulo, Brazil; 4 Department of Microbiology and Immunology, Montana State University, Bozeman, Montana, United States of America; 5 Norris Cotton Cancer Center, Geisel School of Medicine at Dartmouth, Lebanon, New Hampshire, United States of America; 6 Institute for Quantitative Biomedical Sciences, Geisel School of Medicine at Dartmouth, Lebanon, New Hampshire, United States of America; 7 Department of Pathology, Dartmouth-Hitchcock Medical Center, Lebanon, New Hampshire, United States of America; 8 Manchester Fungal Infection Group, School of Biological Sciences, University of Manchester, Manchester, United Kingdom; University of Rochester, UNITED STATES

## Abstract

*Aspergillus fumigatus* is responsible for a disproportionate number of invasive mycosis cases relative to other common filamentous fungi. While many fungal factors critical for infection establishment are known, genes essential for disease persistence and progression are ill defined. We propose that fungal factors that promote navigation of the rapidly changing nutrient and structural landscape characteristic of disease progression represent untapped clinically relevant therapeutic targets. To this end, we find that *A*. *fumigatus* requires a carbon catabolite repression (CCR) mediated genetic network to support *in vivo* fungal fitness and disease progression. While CCR as mediated by the transcriptional repressor CreA is not required for pulmonary infection establishment, loss of CCR inhibits fungal metabolic plasticity and the ability to thrive in the dynamic infection microenvironment. Our results suggest a model whereby CCR in an environmental filamentous fungus is dispensable for initiation of pulmonary infection but essential for infection maintenance and disease progression. Conceptually, we argue these data provide a foundation for additional studies on fungal factors required to support fungal fitness and disease progression and term such genes and factors, *DPFs (disease progression factors)*.

## Author summary

Medical treatment advances such as organ transplants and chemotherapies that suppress the immune system have increased the number of patients susceptible to invasive fungal diseases. The most common filamentous fungus isolated from these infections is the environmental mold, *Aspergillus fumigatus*, the causative agent of invasive aspergillosis (IA). Despite medical intervention, mortality from IA remains high, underscoring the need to understand *A*. *fumigatus* pathogenesis mechanisms to uncover new therapeutic targets and strategies. Here, we show that regulation of central metabolism in *A*. *fumigatus* is critical for infection maintenance and progression of disease. The dysregulation of carbon catabolite repression results in reduced virulence in an animal model at later stages of the infection because of an inability to navigate infection microenvironment dynamics driven in part by oxygen depletion and alterations in nutrient availability. These results likely not only apply to IA, but are broadly applicable to other infection models stressing the need to understand spatiotemporal dynamics of individual microbial infections particularly at the level of metabolism. We propose that microbial genes which support disease progression, in contrast but not mutually exclusive to disease initiation, be termed *DPFs* (disease progression factors) and as such represent a novel class of antimicrobial drug targets.

## Introduction

Microbial pathogenesis is a complex, multifactorial process in which interactions between a microbe and host shape disease development and outcome [1, 2]. While many studies have focused on defining the molecular determinants required for fungal pathogenesis, molecular pathogenesis studies often fail to address the spatial and temporal dynamics of an infection. These dynamics are the result of local changes in nutrients, stressors, and even substrate phase (solid tissue versus liquid environments) that occur over the course of a host-microbe interaction. How fungi navigate these rapidly changing infection dynamics to promote disease is largely unknown and difficult to study with traditional molecular pathogenesis approaches. However, identifying fungal and host factors essential for infection maintenance and disease progression may yield novel and potent therapeutic targets and approaches. With regard to invasive pulmonary aspergillosis (IPA), initiation of disease caused by *A*. *fumigatus* involves conidial deposition into the airways, conidia germination, and subsequent hyphal extension into the lung parenchyma that induces host damage as a result of both fungal growth and inflammation. Many fungal genes and attributes have been identified to be associated with virulence through robust animal model studies [3, 4]. However, patients are typically diagnosed after this initiation phase of the infection when invasion of the lung parenchyma and even vasculature has already occurred. Consequently, it is unclear if fungal virulence factors and attributes identified through animal model virulence studies are relevant at these later stages of the host-pathogen interaction that must be targeted by clinically relevant therapeutics.

It has long been hypothesized that metabolic flexibility significantly contributes to *A*. *fumigatus* virulence; though specific tests of this hypothesis are difficult to achieve due to genetic redundancy in the fungus and the largely uncharacterized dynamic infection microenvironments [5–8]. While the role of several core metabolic pathways has been largely studied in the context of infection initiation (reviewed in [8, 9]), the carbon metabolism regulatory mechanisms required for fungal proliferation within the host as the infection and disease progresses are ill defined. Previously, we observed that established infection microenvironments in murine models of invasive pulmonary aspergillosis (IPA) are depleted in oxygen and contain fungal metabolic by-products such as ethanol [10]. This result was surprising given that *A*. *fumigatus* and related filamentous fungi are obligate aerobes that use glucose as the preferred carbon source and raises important questions about fungal metabolism and bioenergetics throughout the course of an infection. These observations led us to question whether ill-defined mechanisms of *A*. *fumigatus* infection metabolism dynamics are critical for *in vivo* fungal persistence, virulence, and ultimately disease progression.

One mechanism widely employed by microbes to regulate and optimize nutrient acquisition and metabolism is carbon catabolite repression (CCR) [11–13]. Transcriptional regulation of CCR controls central carbon metabolism in many microorganisms and allows prioritization of preferential carbon source usage to yield maximum energy and fitness. The role of CCR in microbial pathogenesis is established in bacteria, and in many species this system plays a role in virulence (reviewed in [14]). For example, in *Streptococcus pyogenes*, the transcriptional regulator CcpA is required for both colonization of the nasopharynx and virulence in invasive disease [15]. Intriguingly, transcriptional regulation of CCR seems dispensable for virulence in human pathogenic yeast based on host survival outcomes. Mig1, the CCR transcriptional regulator in *Candida albicans*, is dispensable for virulence in a systemic murine infection model of candidiasis [16]. Similarly, a *mig1*-null mutant in the basidiomycete yeast *Cryptococcus neoformans* causes wild type levels of disease in a murine inhalation model with no effect on growth within the host though an undefined role for Mig1 in macrophage-yeast interactions was suggested [17]. Yet, given our and other’s previous *in vivo* observations in murine models of IPA, we questioned whether CCR would be dispensable for virulence in an environmental filamentous fungus capable of extreme metabolic flexibility.

To test this hypothesis, we generated a genetic null mutant of the predicted *A*. *fumigatus* CCR transcriptional repressor, CreA. Interrogation of this genetic null mutant *in vivo* and *in vitro* supports the hypothesis that CreA is a CCR transcriptional repressor in *A*. *fumigatus* and surprisingly revealed a crucial role for this system in established infection microenvironments. Our data suggest a model whereby a clinically relevant steroid treatment promotes release of alternative de-repressing carbon sources utilized by *A*. *fumigatus* through CreA independent mechanisms during infection initiation. However, for the infection to proceed and cause life-threatening host damage, dynamic spatial temporal changes in nutrient and oxygen availability at the site of infection drive a requirement for CreA activity in *A*. *fumigatus*. Taken together, our data emphasize the critical importance of spatial temporal dynamics in fungal-host interactions and reveal a novel role for fungal CCR in navigation of infection dynamics and disease progression. We propose that CreA and CCR represent a new class of fungal virulence factors with significant clinical relevance we term DPFs for *disease progression factors*.

## Results

### *A*. *fumigatus* employs a carbon catabolite repression system controlled by CreA

Previously, we observed that oxygen levels within pulmonary fungal lesions are dynamic with oxygen tensions reaching ~1.5% or less by 3 days post inoculation concomitant with detection of ethanol in these animals [10]. Thus, we posited that in the face of this changing nutrient environment, *A*. *fumigatus* must adjust its metabolism to persist and cause additional host damage. To explore this hypothesis, we identified a predicted ortholog of the master fungal CCR transcriptional regulator, CreA, in *Aspergillus fumigatus*. A BLASTP search using the *Aspergillus nidulans* CreA protein sequence as a query against the *A*. *fumigatus* A1163 proteome reveled one homolog of CreA in the *A*. *fumigatus* genome (78% identity; e-value = 4e^-175^), encoded by the gene AFUB_027530. A protein alignment of AfCreA with homologs from *A*. *nidulans* (*Anid*_CreA), *C*. *albicans* (Calb_MIG1; 32% identity), *Cryptococcus neoformans* (Cneo_MIG1; 32% identity), *Trichoderma reesei* (Trees_Cre1; 56% identity) and *Saccharomyces cerevisiae* (Scere_MIG1; 29% identity), reveals little conservation outside of the zinc-finger domains of these 6 CCR transcriptional regulator homologs (S1 Fig). Outside of this region, there is large divergence in the amino acid sequences among these proteins, both in sequence and total length that may suggest the existence of functional differences across species. To study the role of this regulator in *A*. *fumigatus*, we generated genetic null mutant and reconstituted strains of *creA (*Δ*creA* and *creA*^*R*^ respectively; S2 Fig).

**Fig 1:**
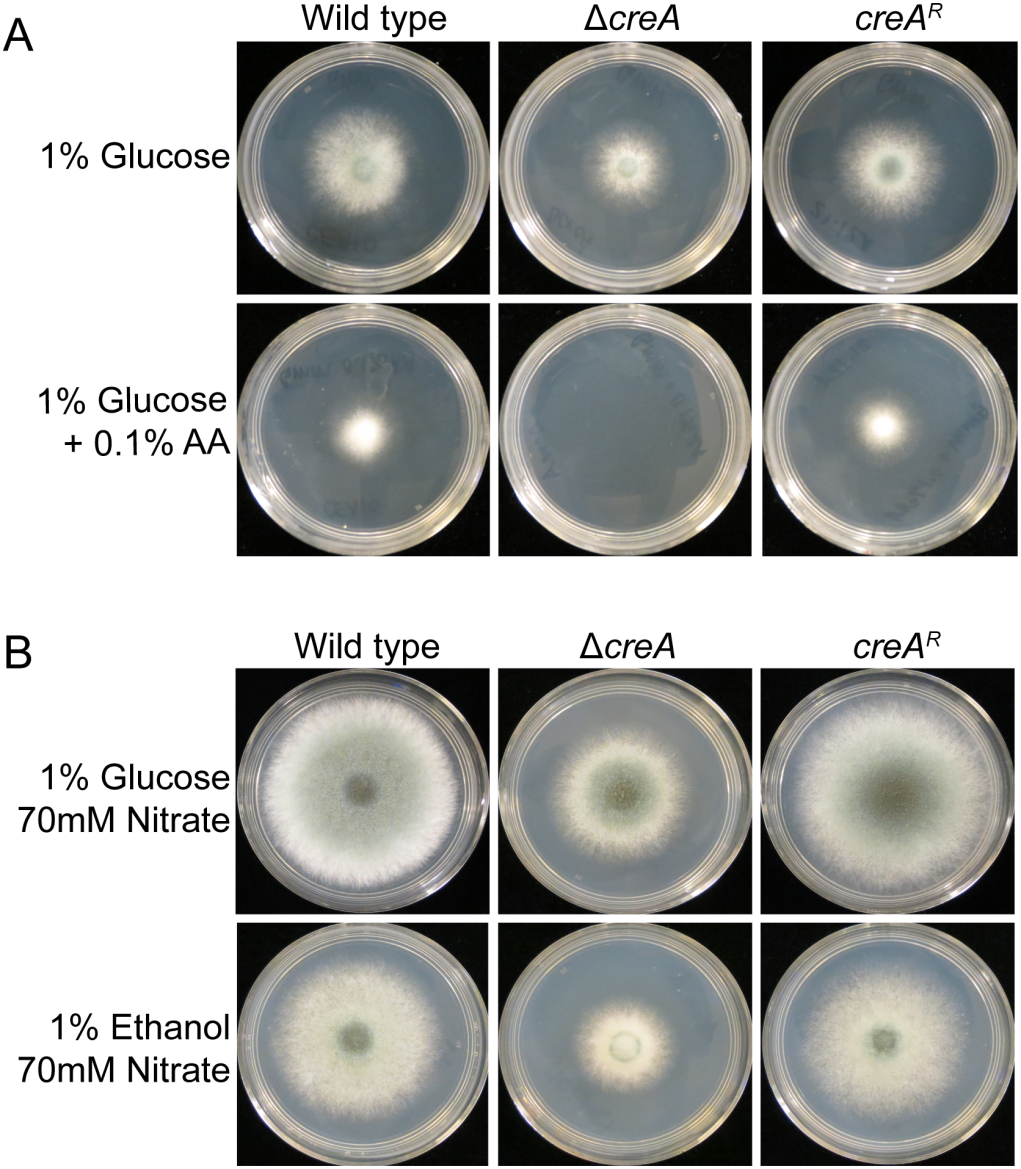
CreA regulates CCR in *A*. *fumigatus*. A) Growth of wild type, Δ*creA*, and *creA*^*R*^ on 1% glucose minimal media with or without 0.1% allyl alcohol (AA) incubated for 48 hours. B) Growth on 1% glucose or 1% ethanol minimal media for 72 hours.

To determine whether CreA controls CCR in *A*. *fumigatus*, we used allyl alcohol as a measure of glucose repression of alcohol dehydrogenases that are well characterized targets of the CCR system in *A*. *nidulans* [18, 19]. Allyl alcohol is metabolized by the *alcA/aldA* gene cluster [20, 21] and broken down into the toxic byproduct, acrolein. When grown on 1% glucose media in the presence of 0.1% allyl alcohol the wild type strain shows a 27% inhibition in radial growth. However, under the same conditions Δ*creA* fails to germinate resulting in 100% growth inhibition (Fig 1A). Re-introduction of *creA* (*creA*^*R*^) restored growth on glucose in the presence of allyl alcohol to wild type levels. As expected, growth of all strains on 1% lactate with 0.1% allyl alcohol was inhibited 100% (S3A Fig), supporting the conclusion that de-repression of glucose-repressed alcohol dehydrogenases is responsible for growth inhibition on allyl alcohol. We also tested the growth of wild type and Δ*creA* on media containing the glucose analog 2-deoxyglucose (2-DG). While the wild type, Δ*creA*, and *creA*^*R*^ strains are not impaired on 1% glucose media with 0.1% 2-DG, the wild type and *creA*^*R*^ strains are partially inhibited on 1% ethanol or 1% lactate with 0.1% 2-DG. However, Δ*creA* growth is significantly less inhibited than the wild type strain, further supporting the conclusion that CreA controls CCR in *A*. *fumigatus* (S3B Fig). Collectively, these results support regulation of CCR by CreA in *A*. *fumigatus*.

### Loss of *creA* reduces fitness in carbon repressing and depressing environments

As expected for a transcriptional regulator of CCR, loss of *creA* results in de-repression of ethanol utilization genes as observed with increased allyl alcohol sensitivity (Fig 1A). Given this role in CCR we tested growth of Δ*creA* on several carbon and nitrogen sources. We observed that fitness of Δ*creA* on solid media is reduced on both repressing (glucose, acetate) and de-repressing carbon sources (ethanol, lactate), as well as on a variety of rich (glutamine, ammonium) and poor nitrogen sources (nitrate, urea) with glucose as a carbon source (Fig 1B; S4A Fig). This result contrasts with CreA homologs in other environmental but non-human pathogenic fungi, such as CRE1 of *T*. *reesei* and Cre-1 of *Neurospora crassa*, where growth defects of the respective CCR mutant are only apparent on particular carbon sources [22, 23]. We also observed comparable growth defects on ethanol, lactate, acetate, and glycerol when glutamine was supplied as the nitrogen source (S4A Fig) and on complete media (S4B Fig). Re-introduction of the *creA* coding sequence (*creA*^*R*^) fully restored fitness of Δ*creA* on all tested conditions. Taken together, these data suggest that CreA is critical for *A*. *fumigatus* fitness on solid substrates in both repressing and de-repressing conditions.

### Regulation of CCR via CreA reveals spatial temporal requirements for *in vivo* fungal fitness and virulence

**Fig 2:**
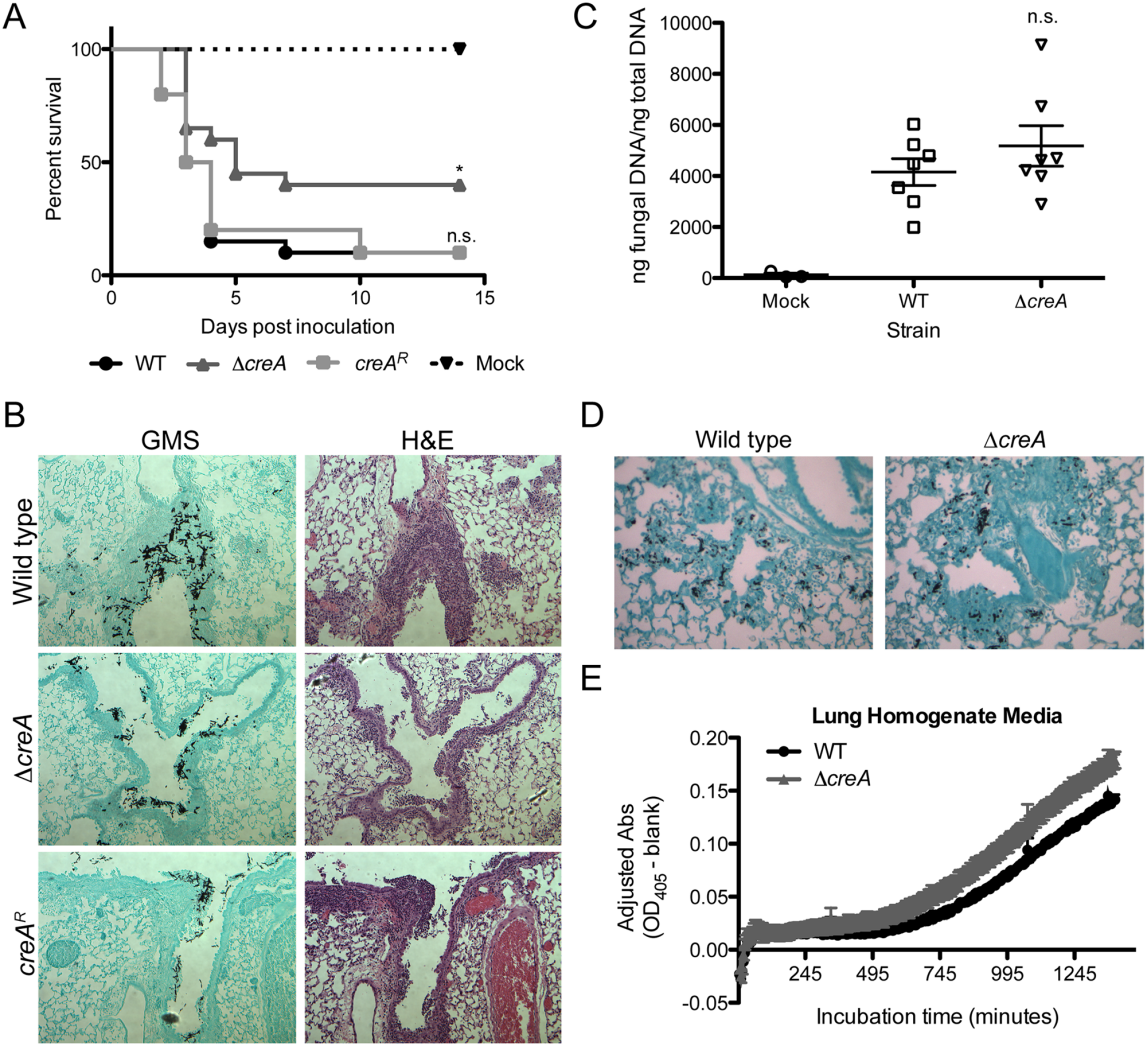
CreA is required for disease progression, but not for establishment of infection in a triamcinolone model of IPA. A) Host survival analysis of fungal strains in a triamcinolone immune suppression model of CD-1 mice inoculated with 2x10^6^ conidia intranasally. n = 20 mice/group from two independent experiments, n = 10 for *creA*^*R*^, n = 4 mice for mock. *p = 0.0217 compared to wild type and p = 0.0425 compared to *creA*^*R*^, n.s. = not significant by Log-rank test. B) GMS and H&E staining of histological sections of lung tissue from CD-1 mice as treated in (A) collected 48 hpi. C) Fungal burden of CD-1 mice as treated in (A), from lungs collected 48 hpi, as measured by qRT-PCR of 18 rDNA region; n = 7 mice/group, n = 3 for mock; n.s. by Wilcoxon rank-sum test as compared to WT; error bars represent SEM. D) GMS staining of histological sections of lung tissue collected 8 hpi from CD-1 mice treated with triamcinolone immune suppression and inoculated with 1x10^7^ conidia. E) Growth of WT and Δ*creA* in lung homogenate media over 24 hours measured by Abs_405_. Data represents mean of six replicates ± SEM.

Given the role of CreA in CCR and growth *in vitro*, we hypothesized that this transcription factor would be critical for virulence. To test the contribution of CreA to *A*. *fumigatus* virulence, we used a triamcinolone-induced immune suppression murine model of IPA [24, 25]. Surprisingly, given the *in vitro* phenotypes of CreA loss, after 2 days post-inoculation, mice inoculated with the wild type, Δ*creA*, or *creA*^*R*^ strains all exhibited symptoms of *A*. *fumigatus* infection. In fact, on day 3 post-inoculation the mortality between all 3 groups was essentially identical with 50% of mice having succumbed to the fungal challenge irrespective of the presence of CreA and a functioning CCR system (Fig 2A).

One potential hypothesis to explain this result is a difference in immunopathogenesis in the presence and absence of CreA. However, no obvious qualitative difference in the inflammatory response was observed between each strain with histopathology. Inflammation observed in all animals by H&E staining of sectioned lung tissue collected 48 hours post inoculation (hpi) was mainly centered upon major airways with extension into the surrounding alveolar parenchyma (Fig 2B). In support of the qualitative data, we observed no significant differences in expression of TNF-α, IL-10 and CXCL1 between mice inoculated with wild type or Δ*creA* conidia, with the exception of increased CXCL1 in response to WT at 72 hpi, a result driven by two animals that had much greater cytokine expression (S5A Fig), indicating that the immune response to each strain is similar.

Consistent with the survival curve and observed similar levels of inflammation, Gomori methenamine silver (GMS) staining indicated that the fungal burden of mice inoculated with wild type and Δ*creA* was indeed similar. Moreover, the amount of tissue involvement in mice inoculated with Δ*creA* both in terms of lesion size and frequency was similar to wild type (Fig 2B). The histopathology data is supported by quantitative measurement of fungal burden using quantification of 18S rDNA 48 hpi that showed no significant difference between wild type and Δ*creA* inoculated mice (Fig 2C). Similarly, we found that Δ*creA* showed no defect in germination at 8 hpi *in vivo*, which further suggests that there is no defect in initiation of infection in the absence of CreA (Fig 2D). These data suggest that the initial *in vivo* airway microenvironment complements the loss of CreA in *A*. *fumigatus* and therefore CCR as mediated by CreA is not necessary for establishment of infection and disease in the lung. To test whether the *in vivo* nutrient environment could complement the loss of CreA as suggested by the *in vivo* data, we tested the growth of Δ*creA* in liquid lung homogenate and observed that growth was similar to the wild type strain (Fig 2E). These data suggest the nutrients available within the steroid treated lung prior to initiation of infection and inflammation are adequate to support the germination and growth of Δ*creA*.

At day 4 post inoculation a marked difference in the infection course emerged between animals inoculated with Δ*creA* compared to the wild type and reconstituted strains. Ninety percent of mice inoculated with the wild-type and reconstituted strain succumbed to the infection at this time point while mice inoculated with Δ*creA* had a significant increase in survival compared to wild type (p = 0.0217) and *creA*^*R*^ (p = 0.0425) that extended out to 14 dpi (Fig 2A). Histopathological analysis of the lungs from the surviving animals inoculated with Δ*creA* show persistent fungal abscesses that are walled off by a thick layer of immune cells that consisted predominantly of neutrophils (S5B Fig). Taken together, these data indicate that transcriptional regulation of filamentous fungal CCR is dispensable for early growth and initiation of disease but essential for virulence and disease progression after infection establishment.

### CreA is critical for fitness in liquid environments containing repressing carbon sources

**Fig 3:**
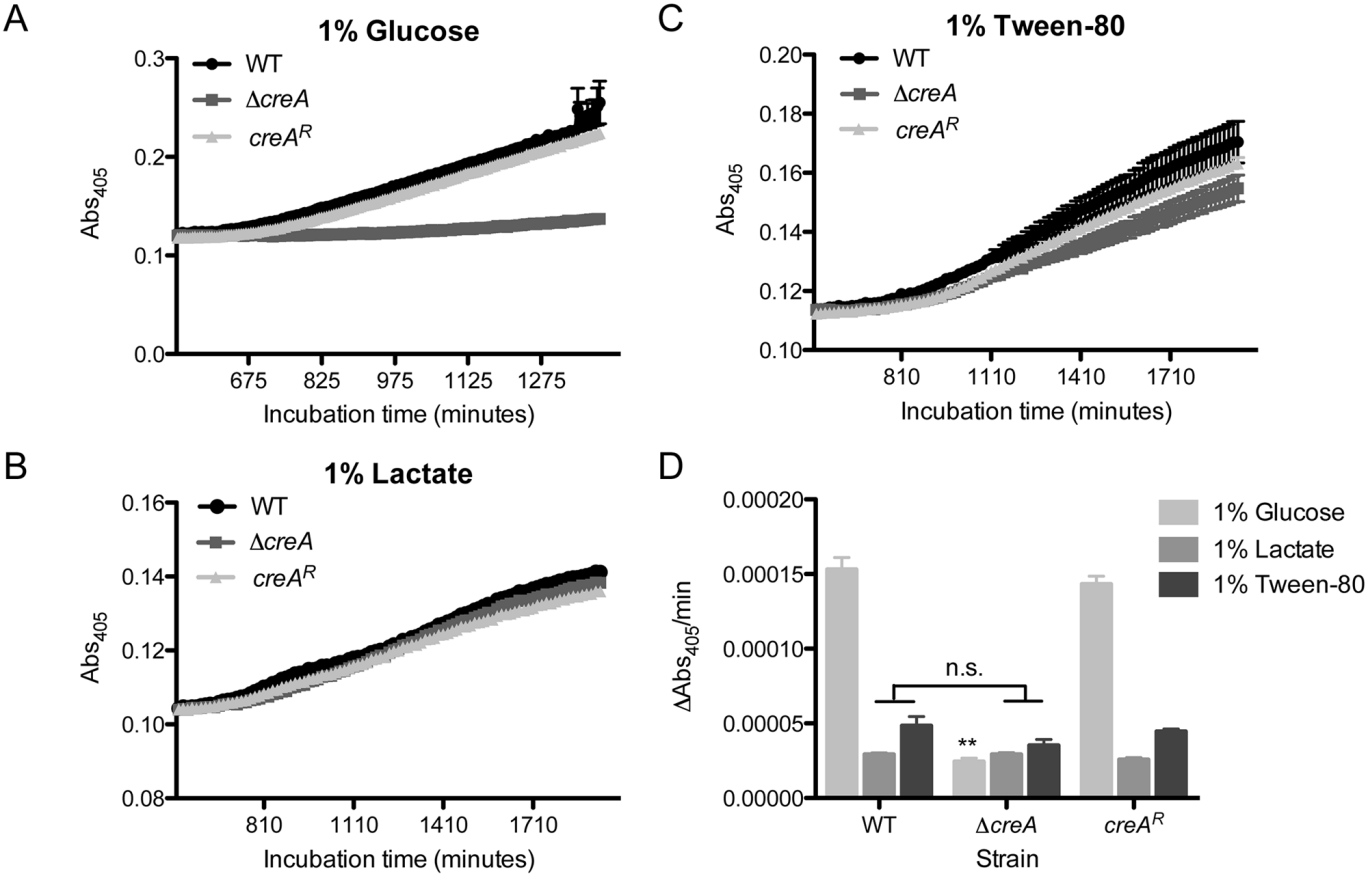
CreA is critical for fitness of *A*. *fumigatus* in liquid media containing repressing carbon sources. Growth curves of WT, Δ*creA* and *creA*^*R*^ in liquid minimal media containing (A) 1% glucose, (B) 1% lactate or (C) 1% Tween-80 as carbon sources. Each curve represents the mean of six replicates ± SEM. D) Quantification of ΔAbs4_05_/min of each condition. **p<0.0001 by unpaired, two tailed t-test as compared to WT of respective condition.

Despite the ability of Δ*creA* to establish infection, the observed temporal virulence defect prompted us to examine the *in vitro* growth phenotypes of this strain more closely to understand underlying mechanisms. In contrast to growth on solid media, Δ*creA* shows a partial growth defect in repressing carbon source liquid media, but not under liquid de-repressing conditions. To test growth initiation in liquid conditions that may better reflect the early infection air-surface liquid interface in the lung, we measured absorbance of cultures grown in 1% glucose, 1% lactate and 1% Tween-80 (Fig 3A, 3B and 3C) and calculated the change in absorbance over time (ΔAbs_405_/min). In 1% glucose, the ΔAbs_405_/min of Δ*creA* is significantly lower than the wild type and *creA*^*R*^. However, under carbon source de-repressing conditions (Tween-80, lactate), the ΔAbs_405_/min of Δ*creA* is strikingly similar to the wild type and *creA*^*R*^ strains (Fig 3D). These results suggest that the initial host environment encountered by *A*. *fumigatus* is enriched for de-repressing carbon sources that promote fungal growth in the absence of transcriptional CCR regulation.

**Fig 4:**
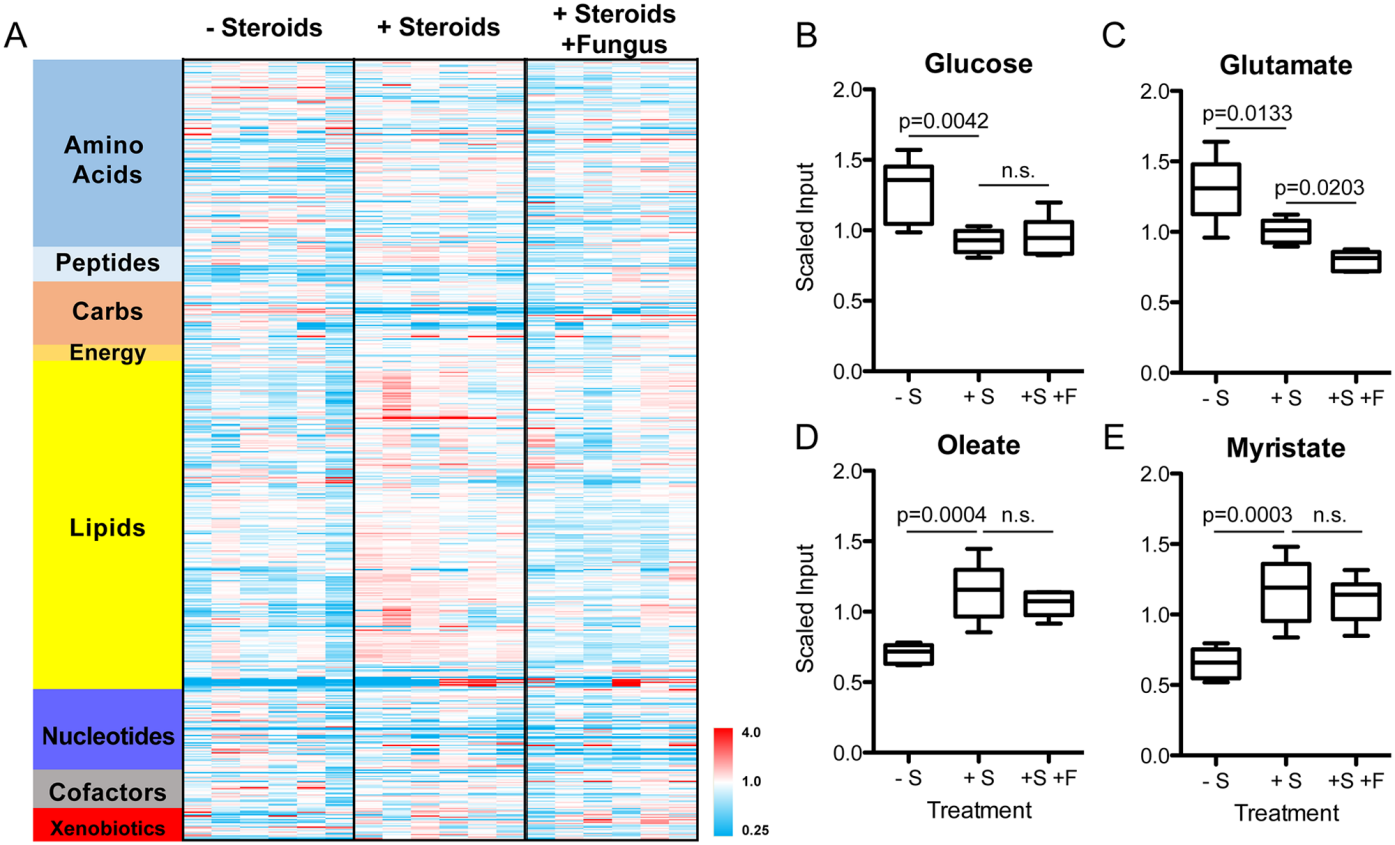
The nutrient landscape of murine lung tissue is complex and dynamic and altered upon treatment with corticosteroids and fungal conidia. A) Heatmap of relative abundance of total detected metabolites from healthy lungs (- Steroids), lungs treated with corticosteroids (+ Steroids) and corticosteroids with fungal conidia (+ Steroids, + Fungus). Each column represents a single outbred CD-1 mouse. B-E) Scaled input values of (B) glucose, (C) glutatmate, (D) oleate and (E) myristate from metabolomics analysis. Significance is calculated with Welch’s Two Sample t-test between indicated groups. Whiskers represent min to max across six biological replicates (6 independent mice).

In support of this hypothesis, a UPLC-MS/MS based steady-state metabolite profile of whole murine lungs of steroid-treated mice versus healthy controls revealed a significant increase in alternative carbon and nitrogen sources upon steroid treatment (Fig 4). Of 630 detected metabolites, 242 metabolites were significantly (p≤0.05) altered in the triamcinolone-treated lungs compared to healthy, untreated controls (Fig 4A, S1 File). Some key findings from this comparison include a significant decrease in glucose (0.71-fold change; p = 0.0042; Fig 4B) and glutamate (0.77-fold change; p = 0.0133; Fig 4C), preferred carbon and nitrogen sources, respectively, for *A*. *fumigatus*, and a concomitant increase in amino acids (leucine, asparagine, valine, methionine, alanine, glutamine, threonine, proline and 5-oxoproline), long chain fatty acids (myristate, arachidate, maragarate, oelate; Fig 4D and 4E), and the alternative nitrogen source, urea. While the steroid treated lungs represent the lung environment first encountered by conidia upon inhalation, we also hypothesized that the environment would change rapidly upon fungal inoculation due to the host immune response. Thus, we also measured the steady-state metabolite profile of steroid-treated animals inoculated with wild type fungal conidia. At 8 hours post inoculation, 168 of 630 detected metabolites significantly (p<0.05) changed between steroid-treated un-inoculated lungs and steroid-treated fungal-inoculated lungs (Fig 4A). While many changes induced by steroid treatment were reversed by inoculation with fungus, glucose remained significantly lower than naïve lungs, and alternative carbon sources such as fatty acids, remained significantly increased (Fig 4B–4D, S1 File). Overall, the nutrient landscape of the lungs at the time of inoculation and during initiation of fungal growth is enriched with alternative carbon sources. These data support the hypothesis that the host environment is complex and dynamic and support the observation that filamentous fungal CCR is dispensable for fungal fitness early in lung infection.

### CreA plays a major role in transcriptional regulation of fungal central carbon metabolism

To gain a deeper understanding of the function of CreA under repressing and de-repressing conditions we used an RNA-Sequencing based approach. Wild type and Δ*creA* were cultured in 1% liquid glucose minimal media (GMM) overnight, then shifted to fresh 1% glucose (repressing) or 1% ethanol (de-repressing) minimal media (EMM) for 2 hours before sample preparation for RNA-sequencing. Importantly, this time point was chosen because growth rate is similar between strains (S4C Fig). Moreover, our previous *in vivo* metabolomics of an IPA murine model revealed significant ethanol levels in the lungs suggesting this alcohol is a potential carbon source seen by *A*. *fumigatus* [10].

In the wild-type strain in de-repressing conditions (EMM), the abundance of 860 transcripts significantly (p<0.05) increased and the abundance of 741 transcripts significantly decreased by at least two-fold (S1 File). As expected, we observed an increase in transcripts of genes involved in the utilization of alternative carbon sources, including the glyoxylate cycle, gluconeogenesis, succinate-fumarate antiporter, aldehyde dehydrogenase and acetyl-coA synthase encoding genes. The induction of these gene indicates generation of anabolic intermediates through gluconeogenesis and subsequent energy production through respiration. As expected, transcript levels of the ethanol utilization alcohol dehydrogenase, *alcA*, increased significantly in EMM, while transcript levels of the fermentation associated alcohol dehydrogenase, *alcC*, decreased [10].

In glucose, loss of CreA resulted in a significant reduction (≥ 2 fold) in transcript levels of 450 genes (p<0.05) and significant increase in transcript levels of 402 genes (S1 File). We verified transcript levels of a subset of genes with qRT-PCR and found that patterns of transcript levels between wild type and Δ*creA* in both glucose and ethanol conditions were consistent between RNA-sequencing and qRT-PCR results (S6 Fig). We applied FunCat analysis to the genes that significantly increase and decrease in Δ*creA* compared to wild type genes using FungiFun2 (S7 Fig) [26]. The categories of increased genes include a large number with predicted functions in transport of metabolites, carbohydrate metabolism, and secondary metabolism. Included in these genes are the alcohol utilization genes *alcA* and *alcS*, the glyoxylate cycle enzymes, isocitrate lyase, *acuD*, and malate synthase, *acuE*, as well as *acuF*, which encodes PEP carboxykinase, and *fbp1*, encoding fructose bisphosphatase aldolase, the rate-limiting steps of gluconeogenesis. Consistent with a role of CreA in regulation of CCR, this transcriptome profile of Δ*creA* suggests that the glyoxylate cycle and gluconeogenesis are active in this strain despite abundant glucose in the environment. The representative categories of decreased genes also include secondary metabolism and c-compound and carbohydrate metabolism in addition to degradation of amino acids. Genes that changed significantly in 1% ethanol between Δ*creA* and wild type were assigned to similar FunCat categories (S1 File; S7 Fig). These data demonstrating significant reductions in mRNA levels in many genes in the absence of CreA may also suggest that CreA has additional roles as a transcriptional activator, but further analyses are required to test this hypothesis. Consequently, these data suggest that loss of *creA* in an environment rich in de-repressing carbon sources such as the steroid treated airway would likely be dispensable for fungal fitness. To further explore the mechanism underlying the observed *in vitro* growth defects of CreA loss we turned to a metabolomics approach.

### CreA regulates fungal bioenergetics and cell wall homeostasis

**Fig 5:**
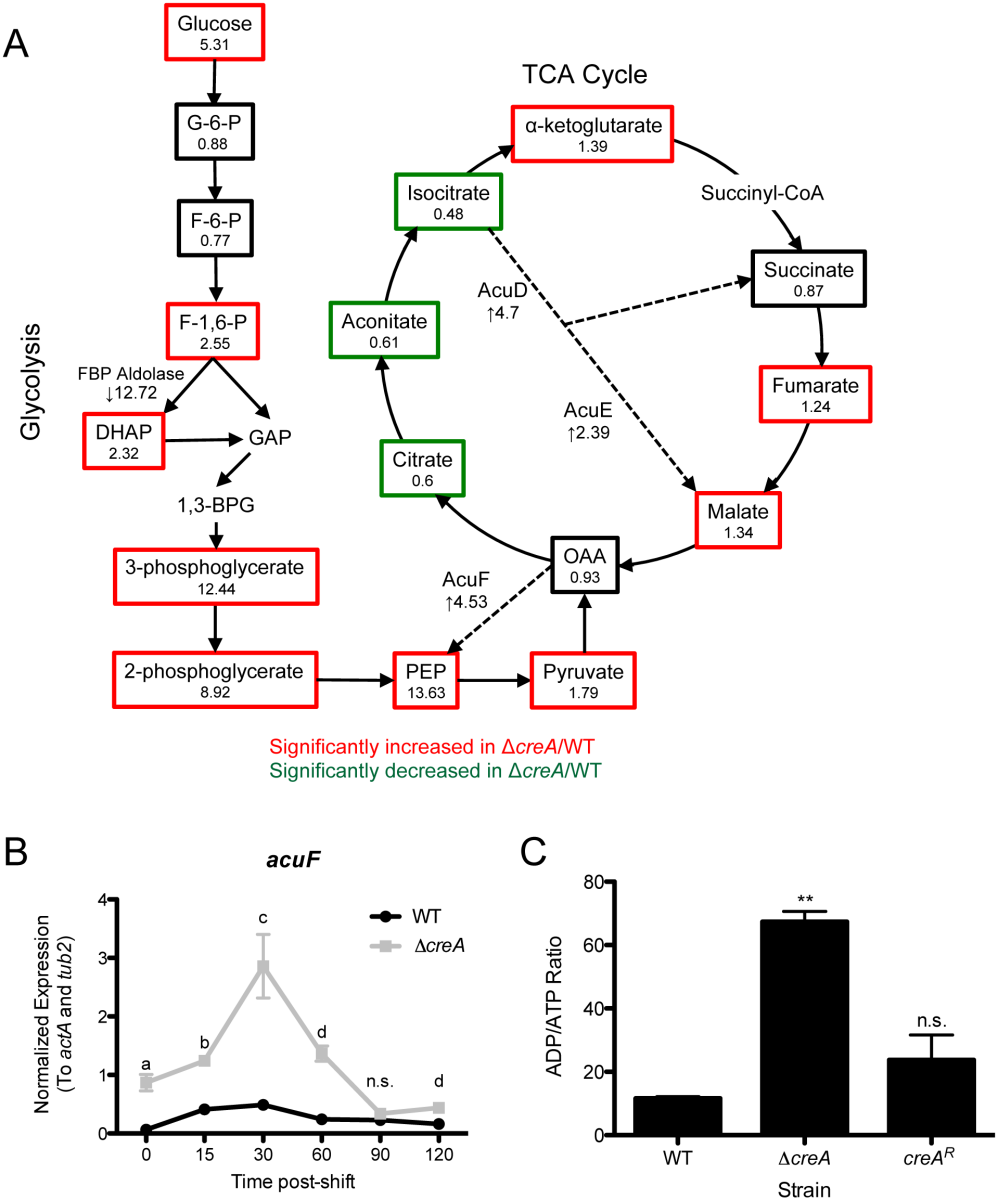
CreA maintains fungal bioenergetics homeostasis. A) Metabolites measured with global metabolomics profiling indicate a perturbation in glucose metabolism and metabolism through the tricarboxylic acid (TCA) cycle. The ratio of each metabolite in Δ*creA*/WT is given under metabolite name. Metabolites that are boxed in red and green are significantly increased and decreased, respectively, in Δ*creA*, and black are not significantly changed (p≤0.05 by Welch’s two-sample t-test). mRNA levels of key metabolic enzymes are given as fold-change in Δ*creA* compared to wild type. B) Expression of *acuF* as measured by qRT-PCR from cultures grown in GMM overnight, then shifted to fresh glucose media with samples taken at indicated time-points post shift. *acuF* expression is normalized to *actA* and *tub2*. ^a^p = 0.0046, ^b^p = 0.0006, ^c^p = 0.0120, ^d^p = 0.001, ^e^p = 0.0054, n.s. = not significant by unpaired, two-tailed t-test as compared to WT of respective time-point. Data represents the mean of three biological replicates ± SEM. C) ADP/ATP ratio of cultures grown in GMM overnight then shifted to fresh GMM for 2 hours. Data represents mean of biological triplicates ± SEM; **p<0.0001, n.s. = not significant by unpaired, two-tailed t-test, as compared to WT.

**Fig 6:**
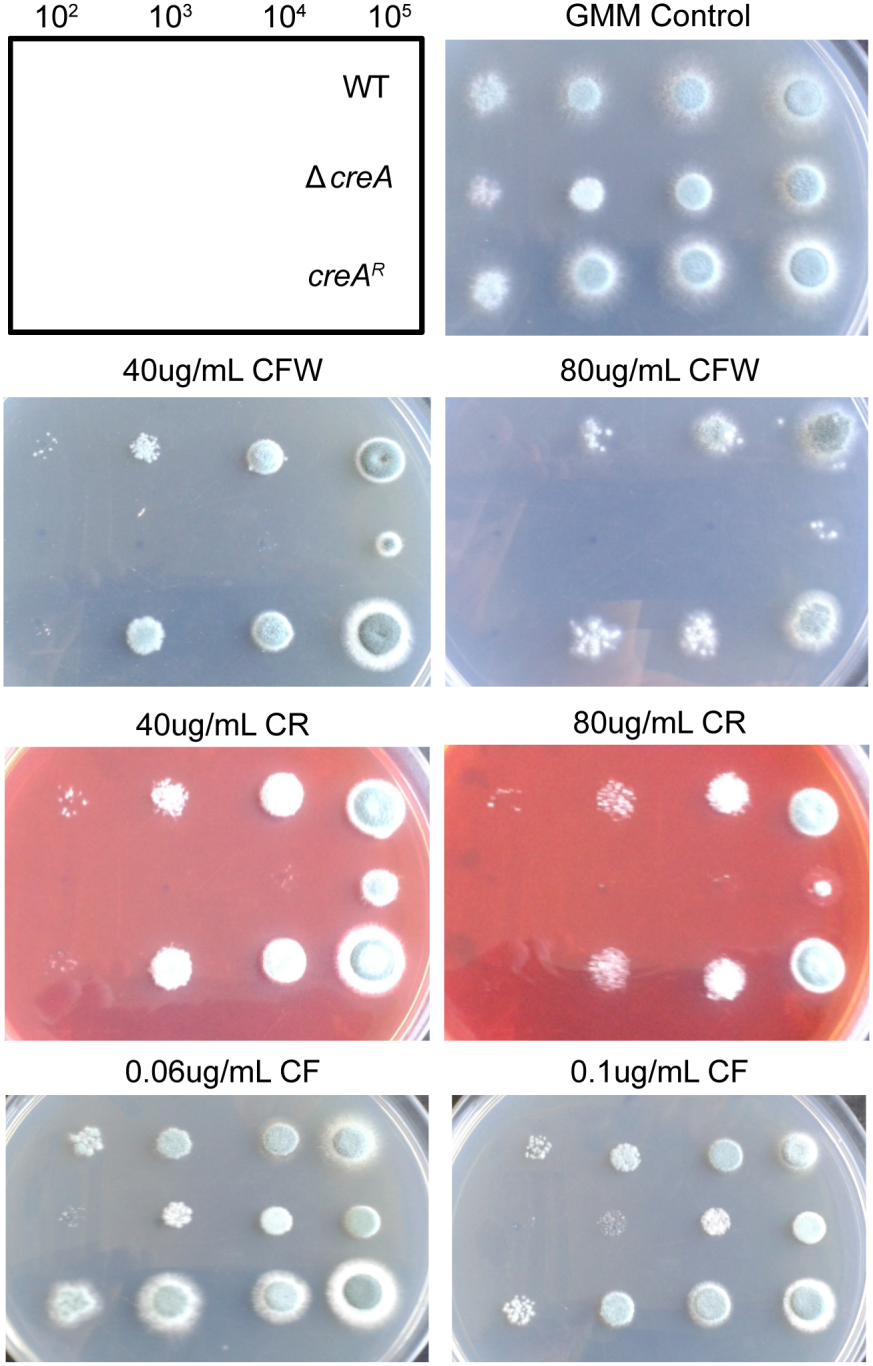
Cell wall homeostasis is perturbed upon loss of CreA. Serial dilutions of WT, Δ*creA* and *creA*^*R*^ on glucose minimal medium with indicated concentrations of Calcoflour white (CFW), Congo Red (CR) or Caspofungin (CF), incubated at 37°C for 48 hours.

Global steady-state metabolomics analysis of Δ*creA* mycelia compared to wild-type revealed striking alterations in glucose metabolism in Δ*creA* (Fig 5A; S1 File). We observed a significant increase (5.31-fold) in the amount of intracellular glucose in Δ*creA*, however no significant changes in metabolites of the early steps of glycolysis (glucose-6-phoshate and fructose-6-phosphate) were observed. In contrast, metabolites of the late steps in glycolysis, including the sugar-phosphates 3-phosphoglycerate (3-PG), 2-phosphoglycerate (2-PG), and phosphenolpyruvate (PEP), were significantly increased in Δ*creA*. In addition, we observed significant changes in tricarboxylic acid (TCA) cycle intermediates. The oxidative portion of the cycle shows significantly altered metabolite levels with citrate, isocitrate and aconitate decreased in the absence of CreA. However, fumarate and malate significantly increase in Δ*creA* compared to wild type (Fig 5A). This pattern of changes in the TCA cycle is consistent with over-expression of isocitrate lyase in *A*. *niger* [27], suggesting that the glyoxylate cycle is active in Δ*creA* despite the presence of glucose. Consistent with this observation, transcript levels of both the predicted isocitrate lyase (*acuD*; 4.7-fold) and malate synthase (*acuE*; 2.39-fold) in the glucose-grown Δ*creA* are significantly increased compared to wild type. Furthermore, transcripts of key gluconeogenic enzymes, PEP carboxykinase (*acuF*; 4.53-fold) and fructose bisphosphatase (*fbp1*; 3.2-fold) are increased in Δ*creA* grown in glucose, suggesting that carbon is being fluxed from the TCA cycle, through the glyoxylate shunt and fed into gluconeogenesis. The over-lap of transcriptomics and metabolomics data sets support the conclusion that CreA is a central regulator of fungal bioenergetics. Given the significant impact of CreA loss on carbon metabolism, we hypothesized that the polysaccharide rich cell wall, closely associated with virulence, would be significantly altered in Δ*creA*. In support of this hypothesis, we observed that Δ*creA* is more sensitive to the cell wall perturbing agents calcoflour white (CFW), congo red (CR) and to a lesser extent caspofungin (CF) when on a glucose containing medium, indicating that cell wall integrity of Δ*creA* is perturbed in these conditions (Fig 6).

To test whether changes in transcript levels of these metabolic enzymes at the time of harvest for RNA-sequencing are a result of true dysregulation or a product of a shift in kinetics of expression following the shift to fresh glucose media, we examined transcript levels of *acuF* over two hours following the shift to fresh glucose. Consistent with perturbation in regulation of these enzymes, we observed transcripts of the gene encoding this enzyme are significantly higher than wild type at nearly all time-points (Fig 5B). Thus, we conclude that loss of CreA results in dysregulation of genes encoding glyoxylate shunt and gluconeogenic enzymes. Moreover, transcript levels of a putative fructose bisphosphate aldolase are 12.72-fold reduced in Δ*creA*, rendering transcript levels of this enzyme nearly absent. We conclude from these data that carbon is being moved through the glyoxylate shunt back into gluconeogenesis despite the already high levels of glucose intracellularly. Consequently, the lack of an FBP-aldolase generates a block in glycolysis/gluconeogenesis, which is responsible for the accumulation of the sugar-phosphate intermediates 3-PG, 2-PG and PEP. The accumulation of these intermediates not only traps cellular carbon, but also lowers available phosphate for cellular bioenergetics and fitness.

In support of this hypothesis, we observed a 5.79-fold increase in the ADP/ATP ratio in Δ*creA* as compared to wild type (Fig 5C). From our metabolomics data, we noted a 5.67-fold increase in the amount of AMP present in Δ*creA* compared to wild type (S1 File). Together, both the increase in AMP and ADP/ATP ratio suggest a defect in mitochondrial output resulting in insufficient cellular bioenergetics. In further support of these data, Δ*creA* is significantly (p<0.0001) more sensitive to the respiratory inhibitors targeting complex II (thenoyltrifluoroacetone; TTFA), complex III (Antimycin A), complex V (Oligomycin A) and the alternative oxidase (Salicylhydroxamic acid; SHAM) when grown on both 1% glucose and 1% ethanol minimal media (S8 Fig). Overall, transcriptomics and metabolomics data suggest that CreA is critical for fungal fitness in environments with repressing carbon sources such as glucose.

### CreA activity supports disease progression in part through promoting fungal fitness in low oxygen infection microenvironments

**Fig 7:**
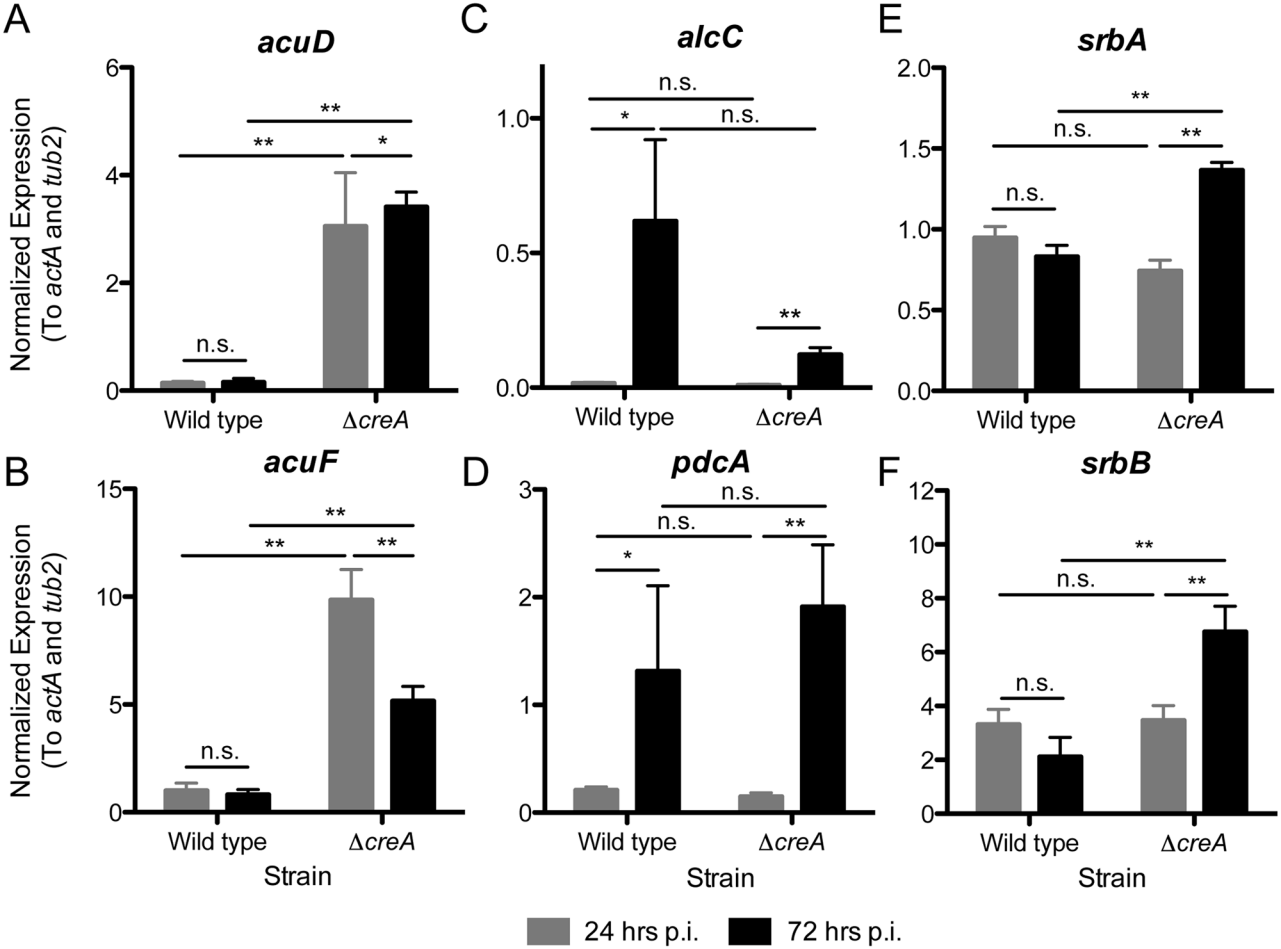
A fungal metabolic shift occurs during infection to favor glycolytic and fermentative metabolism. Expression of (A) *acuD*, (B) *acuF*, (C) *alcC*, (D) *pdcA*, (E) *srbA*, and (F) *srbB* from triamcinolone immune suppressed CD-1 mice 24 and 72 hpi with 5x10^7^ WT or Δ*creA* conidia. Data represents eight biological replicates ± SEM. Gene expression is normalized to *actA* and *tub2*. **p<0.01; *p<0.05; n.s. = not significant by Wilcoxon rank-sum test.

With the observed increase in alternative carbon sources upon steroid treatment, we sought to test the hypothesis that reduced repressing carbon sources early during *A*. *fumigatus* host interaction results in de-repression of CreA-regulated genes. We extracted RNA from triamcinolone treated mice inoculated with fungal conidia 24 and 72 hpi. In support of the model, the CreA-regulated genes, *acuD* and *acuF*, do not increase over time from 24 to 72 hours and the trend was for higher levels early in the infection consistent with increased levels of alternative carbon sources (Fig 7A and 7B). In contrast, the transcript levels of *acuF and acuD*, are significantly increased in Δ*creA* compared to wild type at 24 and 72 hpi, which strongly supports the conclusion that these CreA-target genes identified in our RNA-sequencing data are regulated in part by CreA *in vivo* (Fig 7A and 7D).

To further test our model, we measured the transcript levels of genes expressed in response to severe oxygen depletion, a known characteristic of fungal lesions in the triamcinolone model of IPA [10]. These genes, including the pyruvate decarboxylase, *pdcA*, and alcohol dehydrogenase *alcC*, involved in ethanol production, are significantly increased from 24 hpi to 72 hpi in both wild type and Δ*creA* (Fig 7C and 7D). This increase in hypoxia responsive genes led us to hypothesize that SrbA and SrbB activity, known regulators of the hypoxia response and carbohydrate metabolism in *A*. *fumigatus* [28, 29], increases over the course of infection. However, we did not observe any change in *srbA* mRNA levels from 24 to 72 hpi in the wild type strain perhaps consistent with the absolute requirement of SrbA for *A*. *fumigatus* virulence (Fig 7E). Strikingly, we observed a significant increase in the mRNA levels of *srbA* and *srbB* over the course of the Δ*creA* infection (Fig 7E and 7F). We interpret these data to suggest that Δ*creA* experiences increased reductive stress due to an inability to engage the fungal hypoxia response. Taken together, these data suggest that CreA is critical for maintaining fitness in the low-oxygen environment of established fungal lesions in the lung.

**Fig 8:**
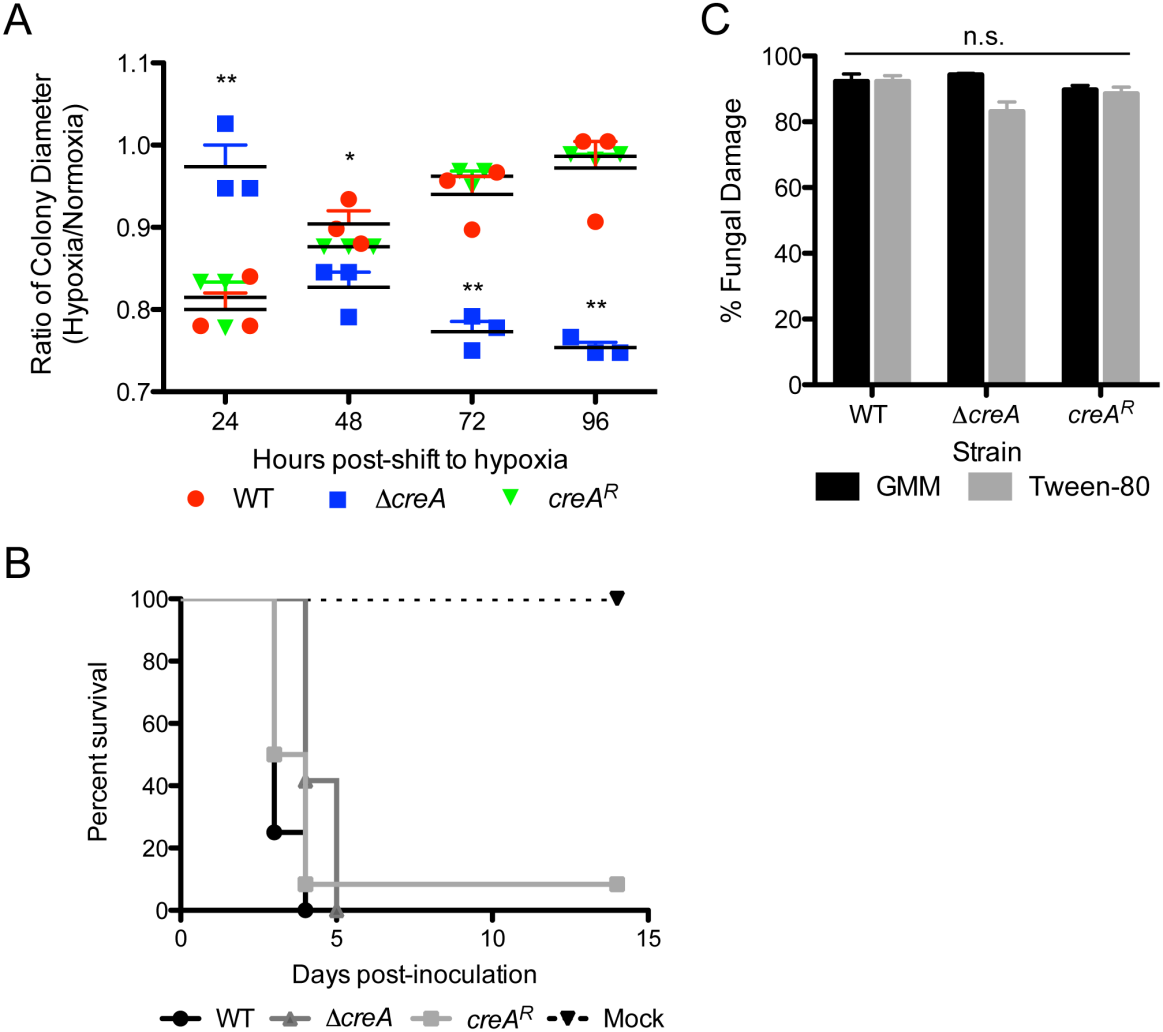
CreA is required for adaptation to changing oxygen environments. A) Ratio of hypoxia to normoxia growth of fungal colonies grown in normoxia for 24 hours, then shifted to hypoxia (0.2% O_2_, 5% CO_2_) for 96 hours. *p<0.05; **p<0.01 by unpaired, two-tailed t-test compared to WT of respective time points. Data represents mean of biological triplicates ± SEM. B) Host survival curve of wild type, Δ*creA* and *creA*^*R*^ in a chemotherapy model of IPA. Mice were treated with 175mg/kg Cyclophosphamide on day -2 and 40mg/kg Kenalog on day -1 then inoculated with 1x10^6^ conidia in 40uL PBS intranasally. n = 12/group, n = 4 for mock. C) Percent fungal damage by bone marrow derived neutrophils of germlings grown in 1% glucose (GMM) or 1% Tween-80 minimal media. Germlings were grown in normoxia conditions, then shifted to hypoxia (0.2% O_2_, 5% CO_2_) upon addition of neutrophils for 2 hours. No significant differences were observed between any group by Wilcoxon rank-sum test.

To test this hypothesis, we measured the growth rate of point inoculated colonies that were germinated in normoxia for 24 hours and then shifted to low oxygen (hypoxia inducing) environment for 96 hours. The ratio of colony growth in hypoxia to normoxia was significantly higher for Δ*creA* compared to wild type and *creA*^*R*^ at the first day post-shift to hypoxia, however, after 48 hours, hypoxia/normoxia growth ratio of Δ*creA* was dramatically lower than wild type, and continued to decrease over time (Fig 8A), which indicates that increased exposure to low oxygen conditions in the presence of a repressing carbon source results in a strong reduction in Δ*creA* growth.

Based on our model of CreA interactions with the host and the inability to adapt to the severe low oxygen conditions of the host environment, we tested the susceptibility of Δ*creA* to killing by neutrophils. We grew wild type, Δ*creA* and *creA*^*R*^ germlings in both repressing (glucose) and de-repressing (Tween-80) conditions, then shifted cultures to a low oxygen environment and added bone marrow derived neutrophils. After 2 hours of incubation with neutrophils, fungal damage was measured by XTT. While we observed no significant difference between damage of wild type and Δ*creA* germlings, or germlings grown in repressing versus de-repressing conditions, all strains, including Δ*creA*, are very sensitive to neutrophil damage under these conditions (Fig 8C).

As our data suggest infection site hypoxia plays an important role in disease progression and a requirement for CreA activity, we utilized the observation that loss of leukocytes reduces the severity of oxygen depletion at the site of fungal infection [10]. Consistent with our data, in this chemotherapy model of IPA, where mice are largely leukopenic, inoculation with Δ*creA* results in 100% mortality by 4 dpi, while wild type results in 100% mortality by 3 dpi (Fig 8B). Therefore, in a host environment with reduced hypoxic and immune cell mediated stress, Δ*creA* is more fit, and produces mortality with near wild type kinetics.

Together, these data suggest that fungal cells are responding to the infection site microenvironment in part through CreA activation which is essential for supporting fungal bioenergetics in the face of oxygen depletion, the dynamic nutrient environment and host immune cell interactions. Therefore, we conclude that CreA represents a novel class of fungal virulence attributes that are dispensable for the initiation of infection but required in established infection microenvironments, which we term disease progression factors (DPFs). Further interrogation of CreA direct genes is thus likely yield additional DPFs in future studies.

### Discussion

Spatial temporal mechanisms of filamentous fungal pathogenesis are under studied. These mechanisms have underappreciated clinical relevance as current antimicrobial therapies largely must thwart pathogens in complex established infection microenvironments to ameliorate disease progression. Yet, in animal models of invasive fungal infections, an emphasis is placed on survival curves with fungal mutants that exhibit marked virulence attenuation from the initiation of infection. Consequently, it is unclear to what extent fungal virulence factors identified from animal model studies mediate infection maintenance and disease progression. In this regard, discovery and critical analysis of regulatory mechanisms essential for fungal fitness and persistence after the initiation of infection and host damage is a promising approach to identify new virulence factors and consequently new and perhaps more clinically relevant drug targets.

Here, we propose that metabolic flexibility allows an environmental microbe, *A*. *fumigatus*, to navigate infection site dynamics and allow disease progression. Our model proposes that during an invasive fungal infection there are at least two distinct fungal metabolic phases driven by changes in nutrient and oxygen availability. For full pathogenic potential and disease progression, cells must undergo metabolic reprogramming to adapt to the changing microenvironments to support proliferation and continued host damage. Our model is not the first to consider this type of adaptation to changing infection dynamics. Saville *et al*. (2003, [30]) used an engineered *C*. *albicans* strain to show that yeast-locked cells could initiate infection, as evidenced by the ability to disseminate to target organs, however, the transition to hyphal morphology was required to cause disease, as measured by murine survival. This study elegantly demonstrates that distinct fungal morphologies are required during different stages of infection and that a morphological transition is essential for *C*. *albicans* virulence.

**Fig 9:**
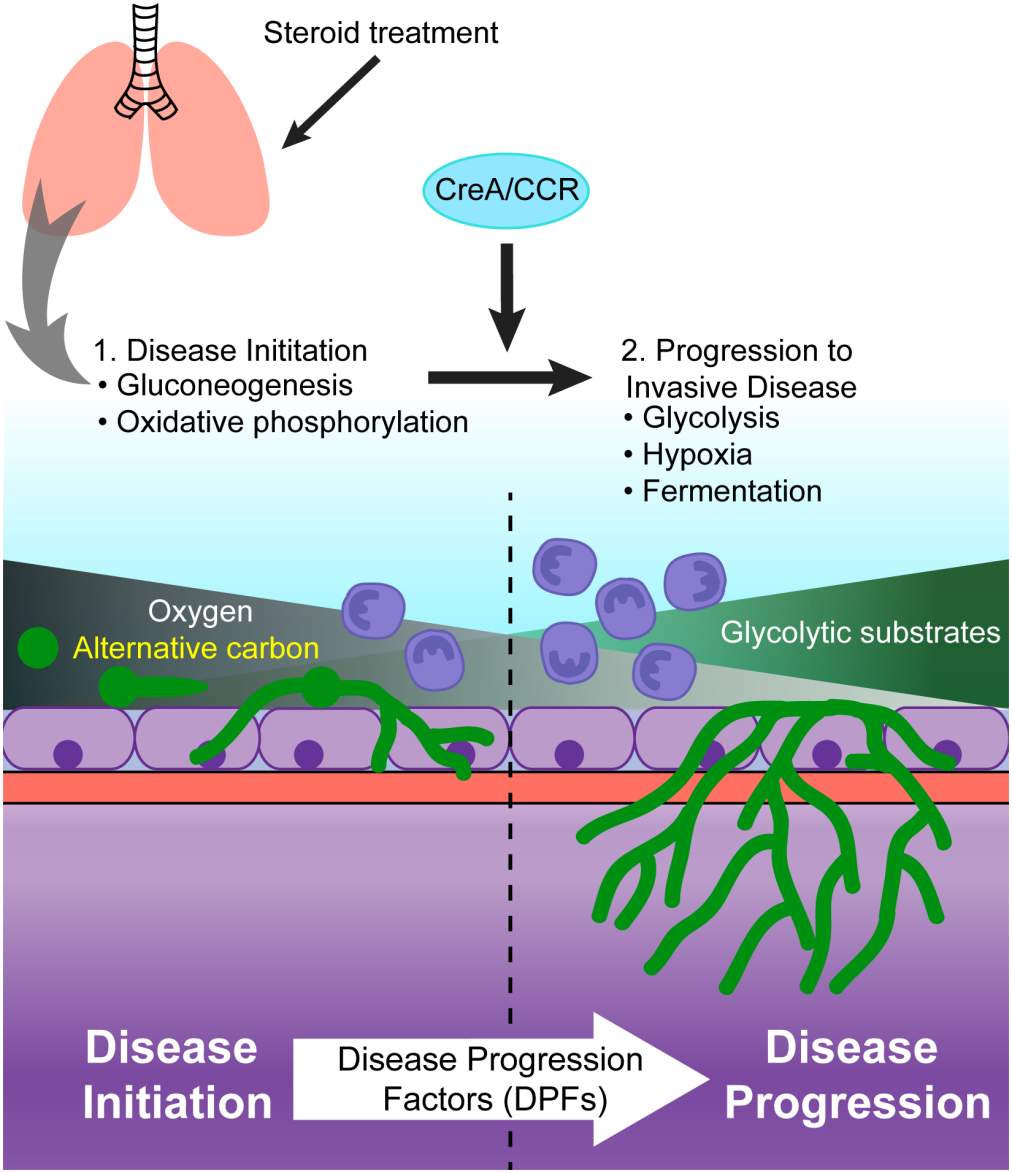
Model for role of CreA in disease progression of invasive aspergillosis. Model of disease progression, where upon infection initiation, the presence of oxygen and alternative carbon sources allows for gluconeogenesis and oxidative phosphorylation. However increased fungal growth and influx of host immune cells results in depletion of local oxygen concentration, shifting metabolism towards glycolytic fermentative metabolism. Fungal metabolic adaptation is required for progression to invasive disease, and this requires CreA to mediate disease progression. We propose the concept of disease progression factors (DPFs) as factors required to navigate the dynamic microenvironments that occur during infection and disease progression.

Our data suggest that early in infection, the steroid treated airway microenvironment contains sufficient gluconeogenic carbon sources and oxygen for fungal conidia germination and growth (Fig 9). In this environment, CCR as mediated by CreA is dispensable. As the infection and disease progresses, carbon source and oxygen availability changes drive the fungal response toward a more glycolytic and hypoxia based metabolism. In this established infection microenvironment, CCR as mediated by CreA significantly contributes to virulence and disease progression. CreA contributes to disease by modulating carbon metabolism and loss of this regulator results in persistent activity of the glyoxylate shunt, which causes metabolic dysregulation in the presences of repressing carbon sources [31]. Importantly, the *creA*-mutant is unable to thrive in the low oxygen environment within the host, as demonstrated through the increase in mRNA levels of the hypoxia response transcription factors, SrbA and SrbB, and the decrease in growth rate upon shift to hypoxia.

A critical consequence of hypoxia in eukaryotic cells is the increase in reducing equivalents. Consequently, the increase in these hypoxia associated gene transcript levels *in vivo* in the absence of CreA strongly suggests a role for CreA in mitigating reductive stress that occurs in these conditions. Although the signals for SREBP activation in mammalian systems are well characterized and depend on cellular sterol levels sensed by the sterol sensor, SCAP [32], SREBP activation mechanism(s) in *A*. *fumigatus* remain unknown [29, 33]. The lack of a SCAP homolog in *A*. *fumigatus*, suggests a distinct mechanism of activation from mammals. An intriguing possibility is regulation of this transcriptional network in response to carbohydrate metabolism and cellular redox status. Support for this hypothesis comes from the observed role of SrbA and SrbB in carbohydrate metabolism and the tight connections between hypoxia and central carbon metabolism in many organisms [34, 35].

Notably, CreA homologs are not required for virulence in the pathogenic yeasts *C*. *albicans* and *C*. *neoformans* as measured by murine mortality [16, 17]. There are several potential reasons for the contrasting results between the yeast systems and our results which highlight differences between the biology and pathogenesis of yeasts and filamentous fungi. First, the models used to test the virulence of the *C*. *albicans* and *C*. *neoformans mig1*-null mutants are significantly different than IPA models. Neither yeast study uses a model that involves the use of corticosteroid immune suppression, which we observed to dramatically alter the metabolite and oxygen landscape of the lungs. Furthermore, *C*. *albicans* murine models often use tail vein delivery of inocula, where yeasts enter the blood, an environment that is very different from the airways in terms of available nutrients and structural landscapes. In particular, the airways contain far less glucose compared to the bloodstream [36–38]. Beyond the models utilized in each study, some observed phenotypes between yeast Mig1 mutants and our CreA mutant are also different, underscoring differences in basal metabolism between these fungi. In *C*. *albicans*, the loss of Mig1 does not affect growth rate in glucose, galactose or glycerol containing media [16] and similarly, growth of *C*. *neoformans* Δ*mig1* shows wild type growth on solid YPD and YES media [17]. Thus, although some observed stress phenotypes between *A*. *fumigatus* and *C*. *neoformans* are similar, the loss of Mig1/CreA has different effects on fungal metabolism across species. For example, in some filamentous fungi, loss of CCR leads to lethality. For example, clean full gene replacements of the CCR transcriptional regulator CreA has not been possible in *Fusarium oxysporum* [39], *Penicillium chrysogenum* [40] and *Colletotrichum gloeosporoides* [41] and results in extremely severe growth defects in *Aspergillus nidulans* [42, 43]. Consequently, the ability to generate a *creA* null mutant in *A*. *fumigatus* and its subsequent phenotypes on de-repressing carbon sources further highlights the diversity of metabolism and bioenergetics regulation in fungi. Whether yeast and other filamentous fungal CCR homologs are DPFs would require additional experiments including assessment of disease in relevant immune compromised animal models.

Although CreA homologs have been shown to be dispensable for virulence in pathogenic yeasts [16, 17], alternative regulation of metabolism through catabolite inactivation, the proteolytic degradation of metabolic enzymes in response to glucose, is important for growth of *C*. *albicans in vivo*. *C*. *albicans* isocitrate lyase (ICL) is subject to transcriptional regulation in response to glucose [44], however, contrary to *S*. *cerevisiae*, ICL is not subject to catabolite inactivation, allowing simultaneous assimilation of glucose and lactate [45]. Consequently, the ability to simultaneously assimilate multiple carbon sources results in higher fungal burden in kidneys during disseminated infection and in feces and kidneys in a gastrointestinal colonization model [46]. Intriguingly, our results suggest that like *C*. *albicans*, *A*. *fumigatus* can integrate both lactate and glucose simultaneously, as inferred from the ability of *A*. *fumigatus* to grow on lactate or ethanol in the presence of 2-DG, suggesting that this fungus is metabolically plastic, an attribute which likely contributes to spatial temporal fitness within the host and ultimately disease maintenance and progression. Therefore, a further understanding of the divergence of the CCR pathway across fungi can yield insights into the mechanistic differences of pathogenicity and virulence across pathogenic fungi for a better understanding of how to treat these infections.

Lastly, we argue that further studies to identify DPFs in pathogenic microbes will identify novel therapeutic targets distinct from existing virulence factors. Though existing virulence factors may certainly be critical for disease progression throughout the course of infection, our data strongly suggest additional factors that may not always be evident from traditional animal model survival curve studies exist and remain to be identified. Moreover, if the metabolic state of the fungus or microbe can be precisely identified during disease progression, *in vitro* systems that induce the same metabolic state are expected to enhance antifungal drug discovery. Moving forward pathogenesis studies should consider the spatial temporal dynamics of infections and leverage developing tools to probe fungal and microbial gene function in established infection microenvironments *in vivo* for high impact therapeutic target identification [47, 48].

## Materials and methods

### Strains and growth conditions

*A*. *fumigatus* CEA17 was used to generate *ΔcreA*. CEA17 is a uracil/uridine auxotrophic mutant of CEA10, therefore, CEA10 was used as the wild type control for all experiments. Strains were stored as conidia in 50% glycerol at -80°C and maintained on 1% glucose minimal media (GMM; 6g/L NaNO_3_, 0.52g/L KCL, 0.52g/L MgSO_4_•7H_2_O, 1.52g/L KH_2_PO_4_ monobasic, 2.2mg/L ZnSO_4_•7H_2_0, 1.1mg/L H_3_BO_3_, 0.5mg/L MnCl_2_•4H_2_O, 0.5mg/L FeSO_4_•7H_2_O, 0.16mg/L CoCl_2_•5H_2_O, 0.16mg/L CuSO_4_•5H_2_O, 0.11mg/L (NH_4_)6Mo_7_O_24_•4H_2_O, 5mg/L Na_4_EDTA, 1% glucose; pH 6.5). All growth assays use the above minimal media with indicated carbon and nitrogen sources; for experiments with alternative nitrogen sources, NaNO_3_ and (NH_4_)6Mo_7_O_24_•4H_2_O are omitted. Solid media was prepared with addition on 1.5% agar (unless otherwise noted) before autoclaving. For all assays, conidia were grown on solid GMM, harvested in 0.01% Tween-80, and counted with a hemacytometer, then diluted to desired concentration in sterile water, unless otherwise noted.

### Strain construction

CreA-null mutants were generated by replacing ~1 kb of the *creA* coding sequence (~1.2 kb) with *A*. *parasiticus pyrG* in the CEA17 background. The replacement construct was generated using overlap extension PCR [49] to join ~1 kb of *creA* 5’ and 3’ UTR with *pyrG*. The resulting construct was used for transformation of protoplasts for selection on media without uracil and uridine supplements. Reconstitution of *creA* in the null mutant was achieved by amplification of the entire *creA* coding sequence including ~1 kb of the flanking 5’ and 3’ regions and the dominant selectable marker *ptrA*, which confers resistance to pyrithiamine. The *creA* coding sequence and *ptrA* were joined with overlap extension PCR, and the linear construct was used to transform Δ*creA* for ectopic expression. The reconstituted strain is denoted as *creA*^*R*^.

Protoplast transformations were carried out as previously described [10, 29]. Briefly, 5-10ug of construct was incubated on ice with protoplasts generated with Lysing Enzyme from *Trichoderma harzianum* (Sigma). PEG/CaCl_2_ solution was added to protoplasts, then incubated at room temperature (RT). Protoplasts were plated on sorbitol stabilized media plates embedded in sorbitol stabilized media agar. Transformants were selected on appropriate media, and screened using PCR to determine if correct integration of the *creA* replacement construct with primers designed to bind within the *pyrG* sequence and outside the integrated construct. Single spores were isolated from positive colonies, and correct integration was confirmed using Southern blotting with the digoxigenin-anti-digoxigenin system (Roche Diagnostics), as previously described [10, 29].

### Growth assays

For alternative carbon and nitrogen plates, the indicated nutrients were added to our minimal media base (nitrogen-free trace elements and salt solution) then plates were inoculated with 1x10^3^ conidia and incubated at 37°C for 72 hours. Allyl alcohol (Sigma) experiments were completed using 1% agar, which was allowed to cool substantially before addition of allyl alcohol to prevent excess evaporation. 1x10^3^ conidia were spotted on plates and incubated at 37°C for 48 hours. Control plates were incubated in a separate incubator, under the same conditions, to prevent inhibition of growth by volatile allyl alcohol. Mitochondrial inhibitors were used in the following concentrations: 0.1mM thenoyltrifluoroacetone (TTFA; Sigma), 15ug/mL Antimycin A (Sigma), 10uM Oligomycin A (Sigma), or 5mM Salicylhydroxamic acid (SHAM; Sigma) in GMM or 1% ethanol minimal media (EMM) agar. Plates were inoculated with 1x10^3^ conidia and incubated at 37°C for 72 hours. Percent inhibition was calculated by measuring radial growth of colonies in the presence of drug, compared to GMM/EMM plates without drug in biological triplicate. All growth assays were completed in biological triplicates.

Liquid growth assays were performed with conidia adjusted to 2x10^4^ conidia in 20uL 0.01% Tween-80 in 96-well dishes, then 180uL of minimal media with indicated carbon source was added to each well. Plates were incubated at 37°C for 7 hours, then Abs_405_ measurements were taken every 15 minutes for the first 24–36 hours of growth with continued incubation at 37°C. Lung homogenate media was prepared as follows: lungs were harvested from healthy CD-1 female mice (20-24g) and homogenized through a 100uM cell strainer in 2mL PBS/lung. Homogenate was diluted 1:4 in sterile PBS, then filter sterilized through 22uM PVDF filters. Each curve represents six technical replicates. ΔAbs405/min was calculated using the linear regression of each individual technical replicate.

Hypoxia shift experiments were performed by inoculation of 1x10^3^ conidia on GMM plates, which were incubated at 37°C for 24 hours in normoxia (21% O_2_, 5%CO_2_) for 24 hours then moved to hypoxia (0.2% O2, 5% CO2; INVOVO2 400 hypoxia chamber [The Baker Company, Sanford, ME]) for 4 days. Radial growth was measured every 24 hours and compared to normoxia controls. Growth is presented as a ratio of hypoxia colony diameter to normoxia colony diameter for three biological replicates.

### ADP/ATP ratio measurement

ADP/ATP ratios were measured from cultures (1x10^6^ conidia/mL) grown in liquid GMM at 37°C for 16 hours with shaking at 250rpm. Fungal tissue was washed with sterile water, and transferred to fresh GMM for 2 additional hours at 37°C with shaking at 250rpm. Tissue was lyophilized, ground with glass beads with a Mini-BeadBeater (BioSpec Products, Inc.). Metabolites were extracted using buffer from ApoSENSOR ADP/ATP Ratio Bioluminescent Assay Kit (K255-200, BioVision) and quantified following manufacturers protocol. Tissue lysates were filtered through Ultracel 10K centrifugal filters (Millipore) prior to processing to remove proteins.

### Isolation of total RNA

Cultures (1x10^6^ conidia/mL) were grown in GMM at 37°C for 16 hours with shaking at 250rpm. Fungal tissue was washed with sterile water, and transferred to fresh media (GMM or EMM) for 2 additional hours at 37°C with shaking at 200rpm. Mycelia were collected with vacuum filtration and immediately frozen with liquid nitrogen. Tissue was lyophilized, then ground with glass beads with a Mini-BeadBeater (BioSpec Products, Inc). RNA was extracted in 1mL of TriSure (Bioline Reagents), then 200uL of chloroform was added, and the aqueous phase was collected after centrifugation. The aqueous phase was in an equal volume of 80% ethanol, then eluted using RNeasy columns (Qiagen) following manufacturer’s instructions.

### Quantitative real-time PCR

5ug of RNA was treated with Ambion Turbo DNAse (Life Technologies) according to manufacturer’s protocol. 500ng of DNAse treated RNA was used for cDNA synthesis with QuantiTech Reverse Transcription kit (Qiagen), then cDNA was diluted 1:5 with ddH_2_O. qRT analysis was performed in 20uL reactions using 2uL of dilute cDNA per reaction with iQ SYBR Green Supermix (BioRad). Gene expression was measured with a BioRad MyiQ Real Time PCR Detection System. For all expression studies, gene expression was normalized to *actA* and *tub2* expression.

### RNA-sequencing

RNA, collected as described above, was analyzed by Qubit and a Fragment Analyzer (Advanced Analytical) for quality control. cDNA libraries were prepared with Illumina’s TruSeq Directional polyA+ library prep kit following the manufacturer’s protocol, with TruSeqLT sequencing adapters/indices. cDNA libraries were multiplexed and loaded at 1.6pM on a NextSeq500 for sequencing. The Illumina raw RNA-seq reads for each replicate were downloaded and then aligned to the *A*. *fumigatus* A1163 genome (CADRE 30) using Tophat v2.1.0 [50] under default parameter settings. Transcripts were then assembled using Cufflinks v2.2.1 [51] and annotated using the A1163 (CADRE 30) GTF file from ENSEMBLFungi. Differential expression analysis was performed for each experimental group separately using DESeq2 [52] and p-values were corrected for multiple testing using Benjamini-Hochberg. NCBI GEO accession number pending.

### Metabolomics sample preparation

Spores were inoculated in GMM at a concentration of 10^6^ conidia/mL and incubated for 16 hours at 37°C with shaking at 250 rpm, then collected with vacuum filtration and washed with sterile water. Fungal tissue was transferred to fresh GMM and further incubated at 37°C, with shaking at 200rpm, for 2 hours. Mycelia were collected with vacuum filtration, washed with sterile water and frozen in liquid nitrogen and stored at -80°C until samples were sent for processing and analysis. Murine metabolomics samples were prepared from whole lungs. CD-1 female mice, 20–24 grams, were administered 40mg/kg Kenalog-10 (Bristol-Myers Squibb) subcutaneously. Lungs of steroid-treated mice were collected 24 hours post injection. For fungal inoculation, immune suppressed mice were inoculated with 2x10^6^ CEA10 conidia in 40uL PBS and lungs were collected 8 hours post inoculation. Healthy controls were given no drug. All lung samples were immediately frozen in liquid nitrogen and stored at -80°C until processing.

### Global metabolomic profiling

Samples were prepared using the automated MicroLab STAR system from Hamilton Company. Several recovery standards were added prior to the first step in the extraction process for QC purposes. To remove protein, dissociate small molecules bound to protein or trapped in the precipitated protein matrix, and to recover chemically diverse metabolites, proteins were precipitated with methanol under vigorous shaking for 2 min (Glen Mills GenoGrinder 2000) followed by centrifugation. The resulting extract was divided into five fractions: two for analysis by two separate reverse phase (RP)/UPLC-MS/MS methods with positive ion mode electrospray ionization (ESI), one for analysis by RP/UPLC-MS/MS with negative ion mode ESI, one for analysis by HILIC/UPLC-MS/MS with negative ion mode ESI, and one sample was reserved for backup. Samples were placed briefly on a TurboVap (Zymark) to remove the organic solvent. The sample extracts were stored overnight under nitrogen before preparation for analysis.

All methods utilized a Waters ACQUITY ultra-performance liquid chromatography (UPLC) and a Thermo Scientific Q-Exactive high resolution/accurate mass spectrometer interfaced with a heated electrospray ionization (HESI-II) source and Orbitrap mass analyzer operated at 35,000 mass resolution. The sample extract was dried then reconstituted in solvents compatible to each of the four methods. Each reconstitution solvent contained a series of standards at fixed concentrations to ensure injection and chromatographic consistency. One aliquot was analyzed using acidic positive ion conditions, chromatographically optimized for more hydrophilic compounds. In this method, the extract was gradient eluted from a C18 column (Waters UPLC BEH C18-2.1x100 mm, 1.7 μm) using water and methanol, containing 0.05% perfluoropentanoic acid (PFPA) and 0.1% formic acid (FA). Another aliquot was also analyzed using acidic positive ion conditions, however it was chromatographically optimized for more hydrophobic compounds. In this method, the extract was gradient eluted from the same afore mentioned C18 column using methanol, acetonitrile, water, 0.05% PFPA and 0.01% FA and was operated at an overall higher organic content. Another aliquot was analyzed using basic negative ion optimized conditions using a separate dedicated C18 column. The basic extracts were gradient eluted from the column using methanol and water, however with 6.5mM Ammonium Bicarbonate at pH 8. The fourth aliquot was analyzed via negative ionization following elution from a HILIC column (Waters UPLC BEH Amide 2.1x150 mm, 1.7 μm) using a gradient consisting of water and acetonitrile with 10mM Ammonium Formate, pH 10.8. The MS analysis alternated between MS and data-dependent MS^n^ scans using dynamic exclusion. The scan range varied slighted between methods but covered 70–1000 m/z.

Peaks were quantified using area-under-the-curve. For studies spanning multiple days, a data normalization step was performed to correct variation resulting from instrument inter-day tuning differences. Essentially, each compound was corrected in run-day blocks by registering the medians to equal one (1.00) and normalizing each data point proportionately. For studies that did not require more than one day of analysis, no normalization is necessary, other than for purposes of data visualization. ‘Scaled Input’ values represent the data rescaled with wild type values set to have a median of one, for ease of visualization. For *in vitro* fungal samples, metabolites were normalized to protein content as determined by a Bradford assay. *In vivo* lung samples represent metabolite content of whole lung without normalization.

### Fungal burden

Outbred female CD-1 mice (Charles River Laboratory, Raleigh, NC), 20–24 grams, were given subcutaneous injections of Kenalog-10 (triamcinolone acetonide, Bristol-Myer Squibb) at 40mg/kg to induce immune-suppression. 24 hours post injection; mice were inoculated with 2x10^6^ fungal spores in 40uL PBS via intranasal inoculation. Mock mice were given 40uL sterile PBS. Mice were sacrificed 48 hours post inoculation and lungs were collected for DNA extraction. Lung tissue was freeze-dried and homogenized with 2.3mm zirconia/silica beads on a Mini-BeadBeater (BioSpec Products, Inc.). Total DNA was extracted using a phenol-chloroform extraction and RNAse treated DNA was used for quantitative PCR, as previously described [24], with primers to amplify 18S region.

### Murine virulence assays

Outbred female mice were immune-suppressed as described above. Inocula of 2x10^6^ CEA10, *ΔcreA*, *creA*^*R*^ conidia were prepared in 40uL PBS and delivered via intranasal inoculation. Mock mice were given 40uL PBS in the absence of fungal spores. For the chemotherapeutic murine model outbred CD-1 female mice, 6 weeks old, were immunosuppressed with intraperitoneal (i.p.) injections of 175mg/kg cyclophosphamide (Baxter Healthcare Corporation) 48 hours before fungal inoculation and subcutaneous (s.c.) injections of 40 mg/kg Kenalog-10 (triamcinolone acetonide, Bristol-Myer Squibb24 hours before fungal inoculation. Mice were inoculated with 1x10^6^ conidia in 40uL PBS intranasally or PBS alone for mock. n = 12 mice/group; n = 4 mice/mock. Mice were housed 4 per cage and had access to food and water *ad libitum*. Mice were monitored for 14 days following challenge with *A*. *fumigatus*. Percent survival was plotted on a Kaplan-Meier curve and a Log-rank test was used to assess statistical significance of the curves.

### Histopathology

CD-1 female mice, 20–24 grams, were immune suppressed as described above. Mice were inoculated with 2x10^6^ conidia of CEA10, Δ*creA*, or *creA*^R^ and lungs were harvested 48 hours post inoculation for histopatholoical sectioning and staining. For early germination studies, immune suppressed mice were inoculated with 1x10^7^ conidia and lungs were harvested 8 hpi for sectioning and staining. Briefly, lungs were perfused with 10% buffered formalin solution upon collection, then fixed in 10% buffered formalin overnight. Lungs were blocked in paraffin, sectioned, and stained with Gömöri methenamine silver (GMS) and hematoxylin and eosin (H&E) stains. Images were obtained with a Zeiss Axioplan 2 imaging microscope (Carl Zeiss Microimaging, Inc.) fitted with Qimiging RETIGA-SRV Fast 1394 RGB camera using Phylum Live 4 imaging software.

### *In vivo* gene expression analysis

Outbred female mice were immune-suppressed as described above and given 5x10^6^ CEA10 or Δ*creA* conidia intranasally in 40uL PBS, 24 hours post injection with steroids. Lungs were harvested 24 and 72 hours post inoculation, and immediately frozen in liquid nitrogen. Lungs were freeze dried and homogenized with 2.3mm zirconia/silica beads on a Mini-BeadBeater (BioSpec Products, Inc.). RNA was extracted using TriSure (Bioline Reagents) then eluted using RNeasy columns (Qiagen) following manufacturer’s instructions. cDNA was synthesized with Qiagen Quantitect Reverse Transcription kit (Qiagen) using 500ng of DNAse treated RNA, and 25ng of resulting cDNA was used in each qRT-PCR reaction. Gene expression was measured with qRT-PCR using BioRad iQ SYBR Green Supermix with a BioRad MyiQ Real Time PCR Detection System (BioRad). To ensure our primers amplified *A*. *fumigatus* genes specifically, we used cDNA from mock mice that had been treated with steroids but given PBS in lieu of fungal spores. All primer pairs tested failed to amplify from mock cDNA.

### Neutrophil killing assay

#### BMDN isolation

Bone-marrow derived neutrophils (BMDNs) were isolated from tibias and femurs of CD-1 females, 6 weeks of age, and cultured for neutrophils in murine neutrophil buffer (HBSS containing 0.1% FBS and 1% glucose). BMDNs were suspended in 3 ml 45% Percoll (GE Healthcare) and isolated from a 30 min 1600x g Percoll gradient in a Sorvall Legend Mach 1.6R benchtop centrifuge, with a BIOshield 600 rotor-75002005 (Thermo Scientific). BMDNs were collected and washed with HBSS before counting as previously described [53].

Fungal cultures of 1x10^5^ conidia were grown in 1mL 1% glucose or 1% Tween-80 minimal media until all strains were germinated to the same extent (time adjusted for slow growth of Δ*creA* in glucose media) in 24-well plates. Media was removed and 1x10^6^ bone marrow derived neutrophils were added to each well in 1mL RPMI, then cultures were shifted to hypoxia (0.2% O_2_, 5% CO_2_) at 37°C for 2 hours. Fungus only (no neutrophils) samples were grown simultaneously for normalization of fungal damage. Following incubation, cells were cold water lysed and the remaining live germlings were quantitated by measuring the OD at 450 nm following a 1 h incubation with 300 μL/well of 0.4 mg/ml XTT (2,3-bis(2- methoxy-4-nitro-5[(sulphenylamino)carbonyl)]-2H-tetrazolium-hydroxide) solution with 50 μg/ml of the electron-coupling agent coenzyme Q. The percent fungal damage was defined by the equation: (1-[A450 of fungi with BMDNs—A450 of BMDNs alone]/[A450 of fungi alone])/100 [54].

### Ethics statement

The Guide for the Care and Use of Laboratory Animals of the National Research Council was strictly followed for all animal experiments. The animal experiment protocols were approved by Institutional Animal Care and Use Committee at Dartmouth College (protocol: cram.ra.1).

## Supporting information

S1 Fig Alignment of CreA homologues across fungal species. Alignment of CreA homolog protein sequences from *A*. *fumigatus (AfCreA)*, *A*. *nidulans (AnCreA)*, *C*. *albicans (CaMig1p)*, *C*. *neoformans (CnMig1p)*, *T*. *reesei (TrCRE1) and S*. *cerevisise (ScMig1p)*. Grey color indicates percent identity across all species, with darker color indicative of higher identity. Alignment was created using Clustal Omega [EMBL-EBI, [55]]. Alignment image was generated using Jalview [56].
(TIF)

S2 Fig Confirmation of *A*. *fumigatus creA*-null mutant and reconstituted strain. A) Schematic of wild type (CEA10) and null-mutant (*ΔcreA*) genomic loci. B) Southern blot of CEA10 (Wild type), *creA* reconstituted (*creA*^*R*^*)* and *creA*-null mutant using BglII digestion and a probe of approximately 1kb of the 5’ UTR of *creA*. CEA10 and *ΔcreA* have the expected bands. *creA*^*R*^ B shows two insertion sites, one which inserted back at the *creA* locus (recombination at 5’ UTR), resulting in loss of the 6174bp band. This strain is used for all subsequent experiments unless otherwise noted, however, both *creA*^*R*^ strains reconstitute all growth phenotypes tested.
(TIF)

S3 Fig Loss of CreA results in increased resistance to 2-DG on alternative carbon sources. A) Growth of strains on 1% lactate minimal media with or without 0.1% allyl alcohol (AA) and incubated for 48 hours. b) Growth inhibition of CEA10, Δ*creA* and *creA*^*R*^ on indicated carbon source with 0.1% 2-deoxyglucose (2-DG) for 72 hours. Data represents mean of biological triplicates ± SEM; ***p<0.0001 by unpaired, two-tailed t-test as compared to WT of respective condition. *creA*^*R*^ strains used for this experiment is *creA*^*R*^ *A*. All assays use 1x10^3^ spore dilutions incubated at 37°C.
(TIF)

S4 Fig Growth of Δ*creA* is reduced on various carbon sources with glutamine supplied as the nitrogen source and on complete media. A) Growth of 1x10^3^ WT, Δ*creA* and *creA*^*R*^ conidia on media containing the indicated carbon and nitrogen sources or (B) Sabouraud Dextrose agar (SDA) for 72 hours at 37°C. C) Biomass (as measured by dry weight) of WT, Δ*creA* and *creA*^*R*^ over 48 hours from cultures of 5x10^5^ conidia/mL, grown at 37°C with shaking at 200rpm.
(TIF)

S5 Fig Late stage infection shows persistence of Δ*creA*. A) Expression of cytokine genes from triamcinolone treated mice, collected 24 or 72 hpi with 5x10^7^ wild type or Δ*creA* conidia. Data represents eight biological replicates ± SEM. Gene expression is normalized to *GAPDH* and *RPL13A*. B) GMS and H&E staining of histological sections of lung tissue from Δ*creA* survivors of triamcinolone model of IPA (see Fig 2A for experimental details), collected 14 days post inoculation.
(TIF)

S6 Fig Verification of RNA-sequencing with qRT-PCR. Expression analysis of A) *alcA*, B) *aldA*, C) *acuD*, which were significantly increased in Δ*creA* compared to wild type in our RNA-sequencing analysis. D) Expression of *dicA*, which did not significantly change in Δ*creA* in the RNA-sequencing analysis. Expression of each gene is normalized to *tub2* and *actA* from cultures grown overnight in 1% glucose, then shifted to 1% glucose (GMM) or 1% ethanol (EMM) minimal media for 2 hours. Error bars represent SEM across four biological replicates. A) **p = 0.0044, *p = 0.0465; B) **p = 0.0015, p = 0.0134; C) *p = 0.0189, **p = 0.0423; D) n.s. = not significant by unpaired, two-tailed t-test as compared to WT of respective conditions.
(TIF)

S7 Fig FunCat analysis of genes significantly changed in Δ*creA* vs. wild type reveals signature of transport functions and carbohydrate metabolism. FunCat category enrichment of genes that significantly increase (A) or decrease (B) by at least 2-fold (p<0.05) in Δ*creA* versus WT for 1% glucose and 1% ethanol conditions. Generated using FungiFun2 [26].
(TIF)

S8 Fig Mitochondrial bioenergetics are perturbed in the absence of CreA. Percent growth inhibition of WT, Δ*creA* and *creA*^*R*^ grown on (A) GMM or (B) EMM in the presence of 0.1mM thenoyltrifluoroacetone (TTFA), 15ug/mL Antimycin A, 10uM Oligomycin A, or 5mM Salicylhydroxamic acid (SHAM) for 72 hours at 37°C. Data represents mean of biological triplicates ± SEM; **p<0.0001; *p = 0.0012; ^#^p = 0.0015 by unpaired, two-tailed t-test as compared to WT of respective conditions. *creA*^*R*^ strain used for this experiment is *creA*^*R*^ *A*.
(TIF)

S1 File Metabolomics of murine lung samples, *in vitro* Δ*creA* metabolomics, significantly changed genes from RNA-sequencing. (XLSX)

## References

[1] Casadevall A, Pirofski LA. The damage-response framework of microbial pathogenesis. Nat Rev Microbiol. 2003;1(1):17–24. 10.1038/nrmicro732 15040176 PMC7097162

[2] Virgin HW. In vivo veritas: pathogenesis of infection as it actually happens. Nat Immunol. 2007;8(11):1143–7. 10.1038/ni1529 17952037

[3] Abad A, Fernandez-Molina JV, Bikandi J, Ramirez A, Margareto J, Sendino J, et al What makes Aspergillus fumigatus a successful pathogen? Genes and molecules involved in invasive aspergillosis. Rev Iberoam Micol. 2010;27(4):155–82. 10.1016/j.riam.2010.10.003 20974273

[4] Kwon-Chung KJ, Sugui JA. Aspergillus fumigatus—what makes the species a ubiquitous human fungal pathogen? PLoS Pathog. 2013;9(12):e1003743 10.1371/journal.ppat.1003743 24348239 PMC3857757

[5] Tekaia F, Latge JP. Aspergillus fumigatus: saprophyte or pathogen? Curr Opin Microbiol. 2005;8(4):385–92. 10.1016/j.mib.2005.06.017 16019255

[6] Rhodes JC. Aspergillus fumigatus: growth and virulence. Med Mycol. 2006;44 Suppl 1:S77–81. 17050423 10.1080/13693780600779419

[7] Brock M. Fungal metabolism in host niches. Curr Opin Microbiol. 2009;12(4):371–6. 10.1016/j.mib.2009.05.004 19535285

[8] Amich J, Krappmann S. Deciphering metabolic traits of the fungal pathogen Aspergillus fumigatus: redundancy vs. essentiality. Front Microbiol. 2012;3:414 10.3389/fmicb.2012.00414 23264772 PMC3525513

[9] Willger SD, Grahl N, Cramer RA Jr. Aspergillus fumigatus metabolism: clues to mechanisms of in vivo fungal growth and virulence. Med Mycol. 2009;47 Suppl 1:S72–9. 19253141 10.1080/13693780802455313 PMC2905159

[10] Grahl N, Puttikamonkul S, Macdonald JM, Gamcsik MP, Ngo LY, Hohl TM, et al In vivo hypoxia and a fungal alcohol dehydrogenase influence the pathogenesis of invasive pulmonary aspergillosis. PLoS Pathog. 2011;7(7):e1002145 10.1371/journal.ppat.1002145 21811407 PMC3141044

[11] Gancedo JM. Carbon catabolite repression in yeast. Eur J Biochem. 1992;206(2):297–313. 1597176 10.1111/j.1432-1033.1992.tb16928.x

[12] Stulke J, Hillen W. Carbon catabolite repression in bacteria. Curr Opin Microbiol. 1999;2(2):195–201. 10.1016/S1369-5274(99)80034-4 10322165

[13] Bailey C, Arst HN Jr. Carbon catabolite repression in Aspergillos nidulans. Eur J Biochem. 1975;51(2):573–7. 168071 10.1111/j.1432-1033.1975.tb03958.x

[14] Gorke B, Stulke J. Carbon catabolite repression in bacteria: many ways to make the most out of nutrients. Nat Rev Microbiol. 2008;6(8):613–24. 10.1038/nrmicro1932 18628769

[15] Shelburne SA 3rd, Keith D, Horstmann N, Sumby P, Davenport MT, Graviss EA, et al A direct link between carbohydrate utilization and virulence in the major human pathogen group A Streptococcus. Proc Natl Acad Sci U S A. 2008;105(5):1698–703. 10.1073/pnas.0711767105 18230719 PMC2234207

[16] Zaragoza O, Rodriguez C, Gancedo C. Isolation of the MIG1 gene from Candida albicans and effects of its disruption on catabolite repression. J Bacteriol. 2000;182(2):320–6. 10629176 10.1128/jb.182.2.320-326.2000 PMC94279

[17] Caza M, Hu G, Price M, Perfect JR, Kronstad JW. The Zinc Finger Protein Mig1 Regulates Mitochondrial Function and Azole Drug Susceptibility in the Pathogenic Fungus Cryptococcus neoformans. mSphere. 2016;1(1). 10.1128/mSphere.00080-15 PMC4863601 27303693

[18] Kulmburg P, Mathieu M, Dowzer C, Kelly J, Felenbok B. Specific binding sites in the alcR and alcA promoters of the ethanol regulon for the CREA repressor mediating carbon catabolite repression in Aspergillus nidulans. Mol Microbiol. 1993;7(6):847–57. 8483416 10.1111/j.1365-2958.1993.tb01175.x

[19] Mathieu M, Felenbok B. The Aspergillus nidulans CREA protein mediates glucose repression of the ethanol regulon at various levels through competition with the ALCR-specific transactivator. EMBO J. 1994;13(17):4022–7. 8076597 10.1002/j.1460-2075.1994.tb06718.x PMC395322

[20] Hynes MJ, Kelly JM. Pleiotropic mutants of Aspergillus nidulans altered in carbon metabolism. Mol Gen Genet. 1977;150(2):193–204. 320455 10.1007/BF00695399

[21] Kelly JM, Hynes MJ. Increased and decreased sensitivity to carbon catabolite repression of enzymes of acetate metabolism in mutants of Aspergillus nidulans. Mol Gen Genet. 1977;156(1):87–92. 23491 10.1007/BF00272256

[22] Nakari-Setala T, Paloheimo M, Kallio J, Vehmaanpera J, Penttila M, Saloheimo M. Genetic modification of carbon catabolite repression in Trichoderma reesei for improved protein production. Appl Environ Microbiol. 2009;75(14):4853–60. 10.1128/AEM.00282-09 19447952 PMC2708423

[23] Sun J, Glass NL. Identification of the CRE-1 cellulolytic regulon in Neurospora crassa. PLoS One. 2011;6(9):e25654 10.1371/journal.pone.0025654 21980519 PMC3183063

[24] Li H, Barker BM, Grahl N, Puttikamonkul S, Bell JD, Craven KD, et al The small GTPase RacA mediates intracellular reactive oxygen species production, polarized growth, and virulence in the human fungal pathogen Aspergillus fumigatus. Eukaryot Cell. 2011;10(2):174–86. 10.1128/EC.00288-10 21183690 PMC3067399

[25] Willger SD, Cornish EJ, Chung D, Fleming BA, Lehmann MM, Puttikamonkul S, et al Dsc orthologs are required for hypoxia adaptation, triazole drug responses, and fungal virulence in Aspergillus fumigatus. Eukaryot Cell. 2012;11(12):1557–67. 10.1128/EC.00252-12 23104569 PMC3536281

[26] Priebe S, Kreisel C, Horn F, Guthke R, Linde J. FungiFun2: a comprehensive online resource for systematic analysis of gene lists from fungal species. Bioinformatics. 2015;31(3):445–6. 10.1093/bioinformatics/btu627 25294921 PMC4308660

[27] Meijer S, Otero J, Olivares R, Andersen MR, Olsson L, Nielsen J. Overexpression of isocitrate lyase-glyoxylate bypass influence on metabolism in Aspergillus niger. Metab Eng. 2009;11(2):107–16. 19271267 10.1016/j.ymben.2008.12.002

[28] Chung D, Barker BM, Carey CC, Merriman B, Werner ER, Lechner BE, et al ChIP-seq and in vivo transcriptome analyses of the Aspergillus fumigatus SREBP SrbA reveals a new regulator of the fungal hypoxia response and virulence. PLoS Pathog. 2014;10(11):e1004487 10.1371/journal.ppat.1004487 25375670 PMC4223079

[29] Willger SD, Puttikamonkul S, Kim KH, Burritt JB, Grahl N, Metzler LJ, et al A sterol-regulatory element binding protein is required for cell polarity, hypoxia adaptation, azole drug resistance, and virulence in Aspergillus fumigatus. PLoS Pathog. 2008;4(11):e1000200 10.1371/journal.ppat.1000200 18989462 PMC2572145

[30] Saville SP, Lazzell AL, Monteagudo C, Lopez-Ribot JL. Engineered control of cell morphology in vivo reveals distinct roles for yeast and filamentous forms of Candida albicans during infection. Eukaryot Cell. 2003;2(5):1053–60. 10.1128/EC.2.5.1053-1060.2003 14555488 PMC219382

[31] Dunn MF, Ramirez-Trujillo JA, Hernandez-Lucas I. Major roles of isocitrate lyase and malate synthase in bacterial and fungal pathogenesis. Microbiology. 2009;155(Pt 10):3166–75. 10.1099/mic.0.030858-0 19684068

[32] Yang T, Espenshade PJ, Wright ME, Yabe D, Gong Y, Aebersold R, et al Crucial step in cholesterol homeostasis: sterols promote binding of SCAP to INSIG-1, a membrane protein that facilitates retention of SREBPs in ER. Cell. 2002;110(4):489–500. 12202038 10.1016/s0092-8674(02)00872-3

[33] Blatzer M, Barker BM, Willger SD, Beckmann N, Blosser SJ, Cornish EJ, et al SREBP coordinates iron and ergosterol homeostasis to mediate triazole drug and hypoxia responses in the human fungal pathogen Aspergillus fumigatus. PLoS Genet. 2011;7(12):e1002374 10.1371/journal.pgen.1002374 22144905 PMC3228822

[34] Vodisch M, Scherlach K, Winkler R, Hertweck C, Braun HP, Roth M, et al Analysis of the Aspergillus fumigatus proteome reveals metabolic changes and the activation of the pseurotin A biosynthesis gene cluster in response to hypoxia. J Proteome Res. 2011;10(5):2508–24. 10.1021/pr1012812 21388144 PMC3091480

[35] Barker BM, Kroll K, Vodisch M, Mazurie A, Kniemeyer O, Cramer RA. Transcriptomic and proteomic analyses of the Aspergillus fumigatus hypoxia response using an oxygen-controlled fermenter. BMC Genomics. 2012;13:62 10.1186/1471-2164-13-62 22309491 PMC3293747

[36] Philips BJ, Meguer JX, Redman J, Baker EH. Factors determining the appearance of glucose in upper and lower respiratory tract secretions. Intensive Care Med. 2003;29(12):2204–10. 10.1007/s00134-003-1961-2 14647890

[37] Baker EH, Clark N, Brennan AL, Fisher DA, Gyi KM, Hodson ME, et al Hyperglycemia and cystic fibrosis alter respiratory fluid glucose concentrations estimated by breath condensate analysis. J Appl Physiol (1985). 2007;102(5):1969–75. 17303703 10.1152/japplphysiol.01425.2006

[38] Egi M, Bellomo R, Stachowski E, French CJ, Hart GK, Hegarty C, et al Blood glucose concentration and outcome of critical illness: the impact of diabetes. Crit Care Med. 2008;36(8):2249–55. 10.1097/CCM.0b013e318181039a 18664780

[39] Jonkers W, Rep M. Mutation of CRE1 in Fusarium oxysporum reverts the pathogenicity defects of the FRP1 deletion mutant. Mol Microbiol. 2009;74(5):1100–13. 10.1111/j.1365-2958.2009.06922.x 19912543

[40] Cepeda-García C, Domínguez-Santos R, García-Rico RO, García-Estrada C, Cajiao A, Fierro F, et al Direct involvement of the CreA transcription factor in penicillin biosynthesis and expression of the pcbAB gene in Penicillium chrysogenum. Applied Microbiology and Biotechnology. 2014;98(16):7113–24. 10.1007/s00253-014-5760-1 24818689

[41] Bi F, Barad S, Ment D, Luria N, Dubey A, Casado V, et al Carbon regulation of environmental pH by secreted small molecules that modulate pathogenicity in phytopathogenic fungi. Molecular Plant Pathology. 2015:n/a–n/a. 10.1111/mpp.12355 PMC6638356 26666972

[42] Dowzer CE, Kelly JM. Analysis of the creA gene, a regulator of carbon catabolite repression in Aspergillus nidulans. Mol Cell Biol. 1991;11(11):5701–9. 1922072 10.1128/mcb.11.11.5701-5709.1991 PMC361941

[43] Shroff RA, O'Connor SM, Hynes MJ, Lockington RA, Kelly JM. Null alleles of creA, the regulator of carbon catabolite repression in Aspergillus nidulans. Fungal Genet Biol. 1997;22(1):28–38. 10.1006/fgbi.1997.0989 9344629

[44] Rodaki A, Bohovych IM, Enjalbert B, Young T, Odds FC, Gow NA, et al Glucose promotes stress resistance in the fungal pathogen Candida albicans. Mol Biol Cell. 2009;20(22):4845–55. 10.1091/mbc.E09-01-0002 19759180 PMC2777113

[45] Sandai D, Yin Z, Selway L, Stead D, Walker J, Leach MD, et al The evolutionary rewiring of ubiquitination targets has reprogrammed the regulation of carbon assimilation in the pathogenic yeast Candida albicans. MBio. 2012;3(6). 10.1128/mBio.00495-12 PMC3520108 23232717

[46] Childers DS, Raziunaite I, Mol Avelar G, Mackie J, Budge S, Stead D, et al The Rewiring of Ubiquitination Targets in a Pathogenic Yeast Promotes Metabolic Flexibility, Host Colonization and Virulence. PLoS Pathog. 2016;12(4):e1005566 10.1371/journal.ppat.1005566 27073846 PMC4830568

[47] Sasse A, Hamer SN, Amich J, Binder J, Krappmann S. Mutant characterization and in vivo conditional repression identify aromatic amino acid biosynthesis to be essential for Aspergillus fumigatus virulence. Virulence. 2016;7(1):56–62. 10.1080/21505594.2015.1109766 26605426 PMC4871646

[48] Cramer RA. In vivo veritas: Aspergillus fumigatus proliferation and pathogenesis—conditionally speaking. Virulence. 2016;7(1):7–10. 10.1080/21505594.2015.1134074 26695225 PMC4871685

[49] Szewczyk E, Nayak T, Oakley CE, Edgerton H, Xiong Y, Taheri-Talesh N, et al Fusion PCR and gene targeting in Aspergillus nidulans. Nat Protoc. 2006;1(6):3111–20. 10.1038/nprot.2006.405 17406574

[50] Trapnell C, Pachter L, Salzberg SL. TopHat: discovering splice junctions with RNA-Seq. Bioinformatics. 2009;25(9):1105–11. 10.1093/bioinformatics/btp120 19289445 PMC2672628

[51] Trapnell C, Roberts A, Goff L, Pertea G, Kim D, Kelley DR, et al Differential gene and transcript expression analysis of RNA-seq experiments with TopHat and Cufflinks. Nat Protoc. 2012;7(3):562–78. 10.1038/nprot.2012.016 22383036 PMC3334321

[52] Love MI, Huber W, Anders S. Moderated estimation of fold change and dispersion for RNA-seq data with DESeq2. Genome Biol. 2014;15(12):550 10.1186/s13059-014-0550-8 25516281 PMC4302049

[53] Shepardson KM, Jhingran A, Caffrey A, Obar JJ, Suratt BT, Berwin BL, et al Myeloid derived hypoxia inducible factor 1-alpha is required for protection against pulmonary Aspergillus fumigatus infection. PLoS pathogens. 2014;10(9):e1004378 Epub 2014/09/26. 10.1371/journal.ppat.1004378 25255025 PMC4177996

[54] Shepardson KM, Ngo LY, Aimanianda V, Latge JP, Barker BM, Blosser SJ, et al Hypoxia enhances innate immune activation to Aspergillus fumigatus through cell wall modulation. Microbes and infection / Institut Pasteur. 2013;15(4):259–69. Epub 2012/12/12. 10.1016/j.micinf.2012.11.010 PMC3723392 23220005

[55] Sievers F, Wilm A, Dineen D, Gibson TJ, Karplus K, Li W, et al Fast, scalable generation of high-quality protein multiple sequence alignments using Clustal Omega. Mol Syst Biol. 2011;7:539 10.1038/msb.2011.75 21988835 PMC3261699

[56] Waterhouse AM, Procter JB, Martin DM, Clamp M, Barton GJ. Jalview Version 2—a multiple sequence alignment editor and analysis workbench. Bioinformatics. 2009;25(9):1189–91. 10.1093/bioinformatics/btp033 19151095 PMC2672624

