# Supplementary figures and images for: Filamentous fungal carbon catabolite repression supports metabolic plasticity and stress responses essential for disease progression
Source: PLoS Pathog. 2017 Apr 19;13(4):e1006340. doi: 10.1371/journal.ppat.1006340 (PMC5411099; doi:10.1371/journal.ppat.1006340)

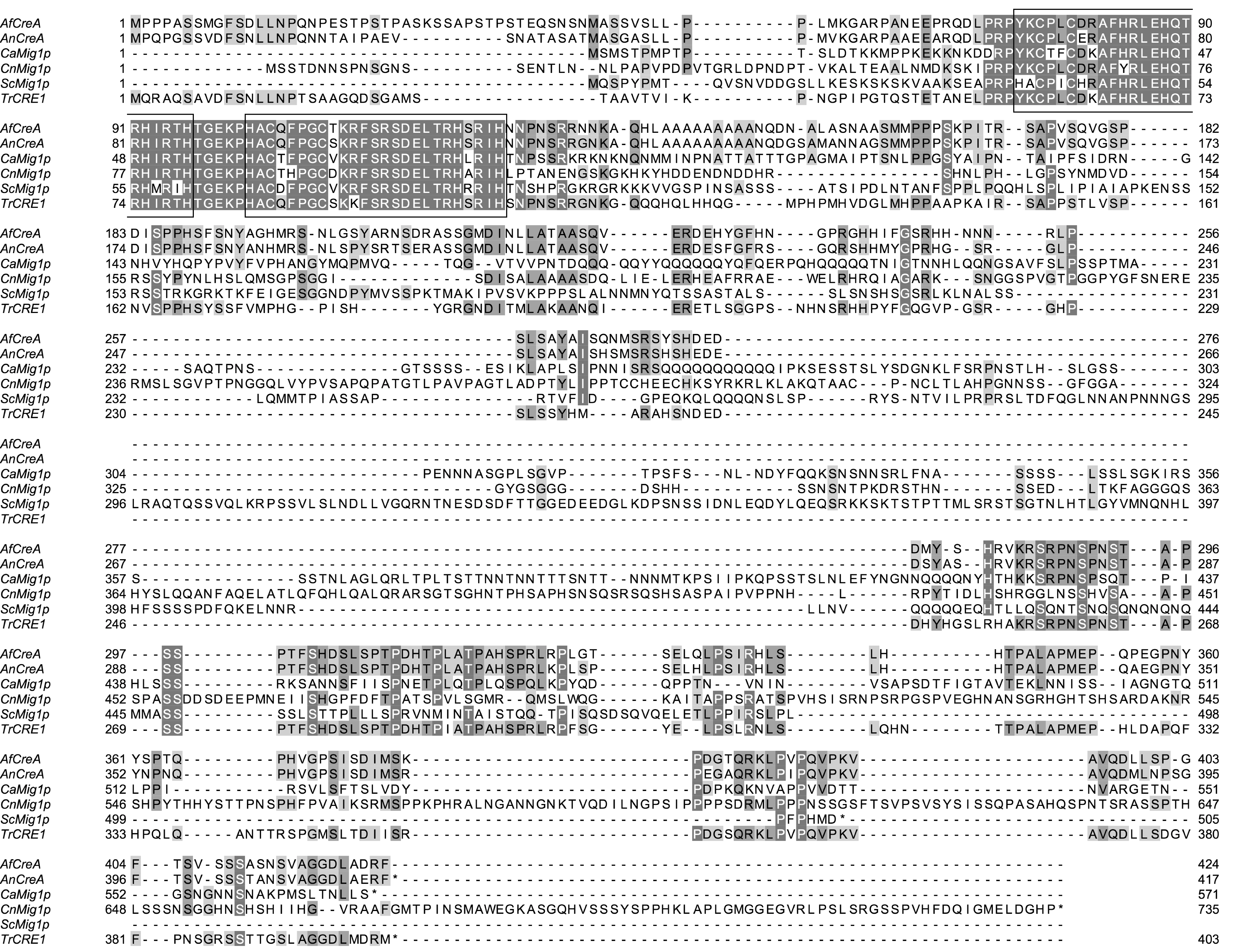

Supplement: S1 Fig — Alignment of CreA homolog protein sequences from A. fumigatus (AfCreA), A. nidulans (AnCreA), C. albicans (CaMig1p), C. neoformans (CnMig1p), T. reesei (TrCRE1) and S. cerevisise (ScMig1p). Grey color indicates percent identity across all species, with darker color indicative of higher identity. Alignment was created using Clustal Omega [EMBL-EBI, [55]]. Alignment image was generated using Jalview [56]. (TIF) [file ppat.1006340.s001.tif]

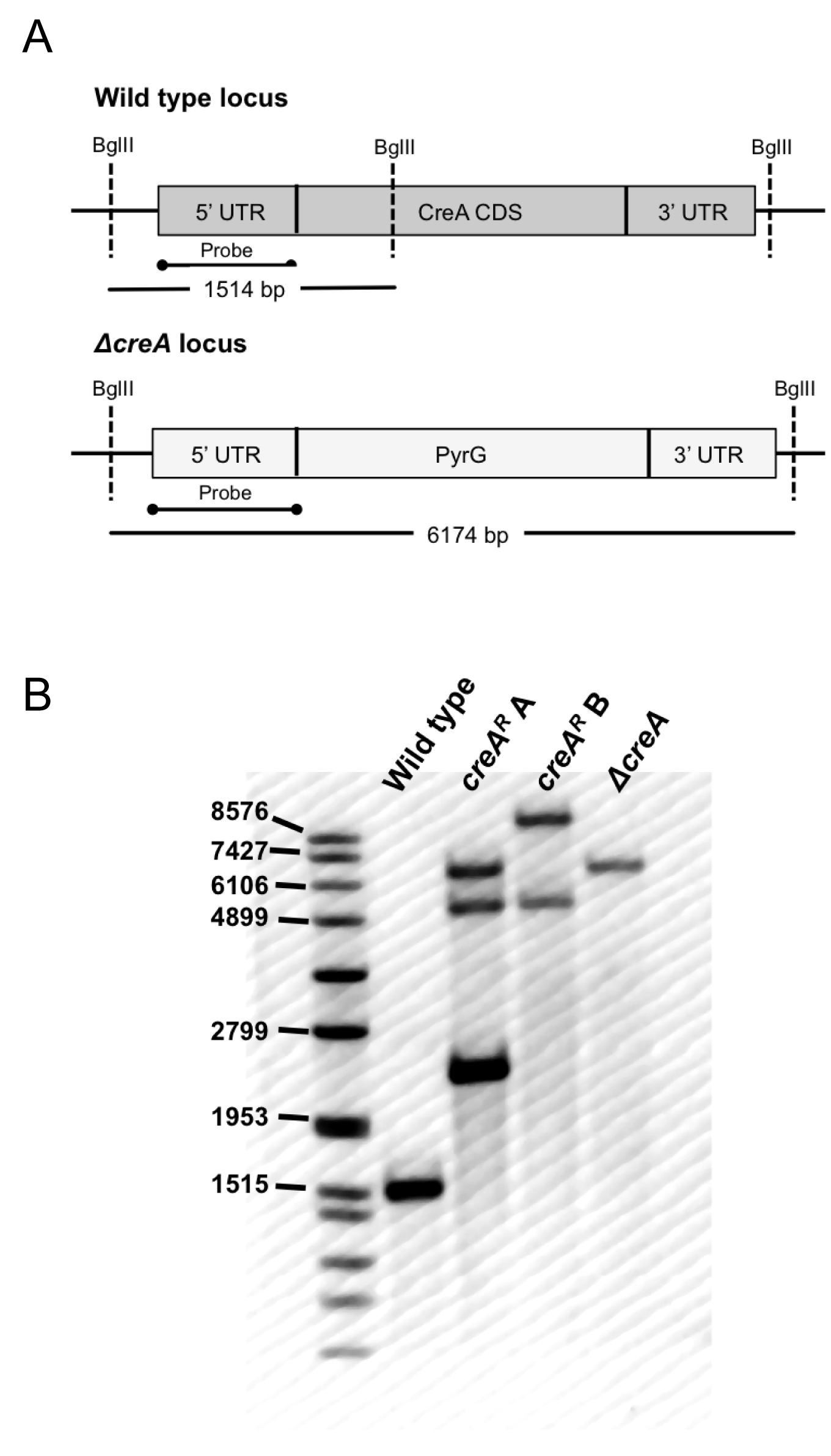

Supplement: S2 Fig — A) Schematic of wild type (CEA10) and null-mutant (ΔcreA) genomic loci. B) Southern blot of CEA10 (Wild type), creA reconstituted (creAR) and creA-null mutant using BglII digestion and a probe of approximately 1kb of the 5’ UTR of creA. CEA10 and ΔcreA have the expected bands. creAR B shows two insertion sites, one which inserted back at the creA locus (recombination at 5’ UTR), resulting in loss of the 6174bp band. This strain is used for all subsequent experiments unless otherwise noted, however, both creAR strains reconstitute all growth phenotypes tested. (TIF) [file ppat.1006340.s002.tif]

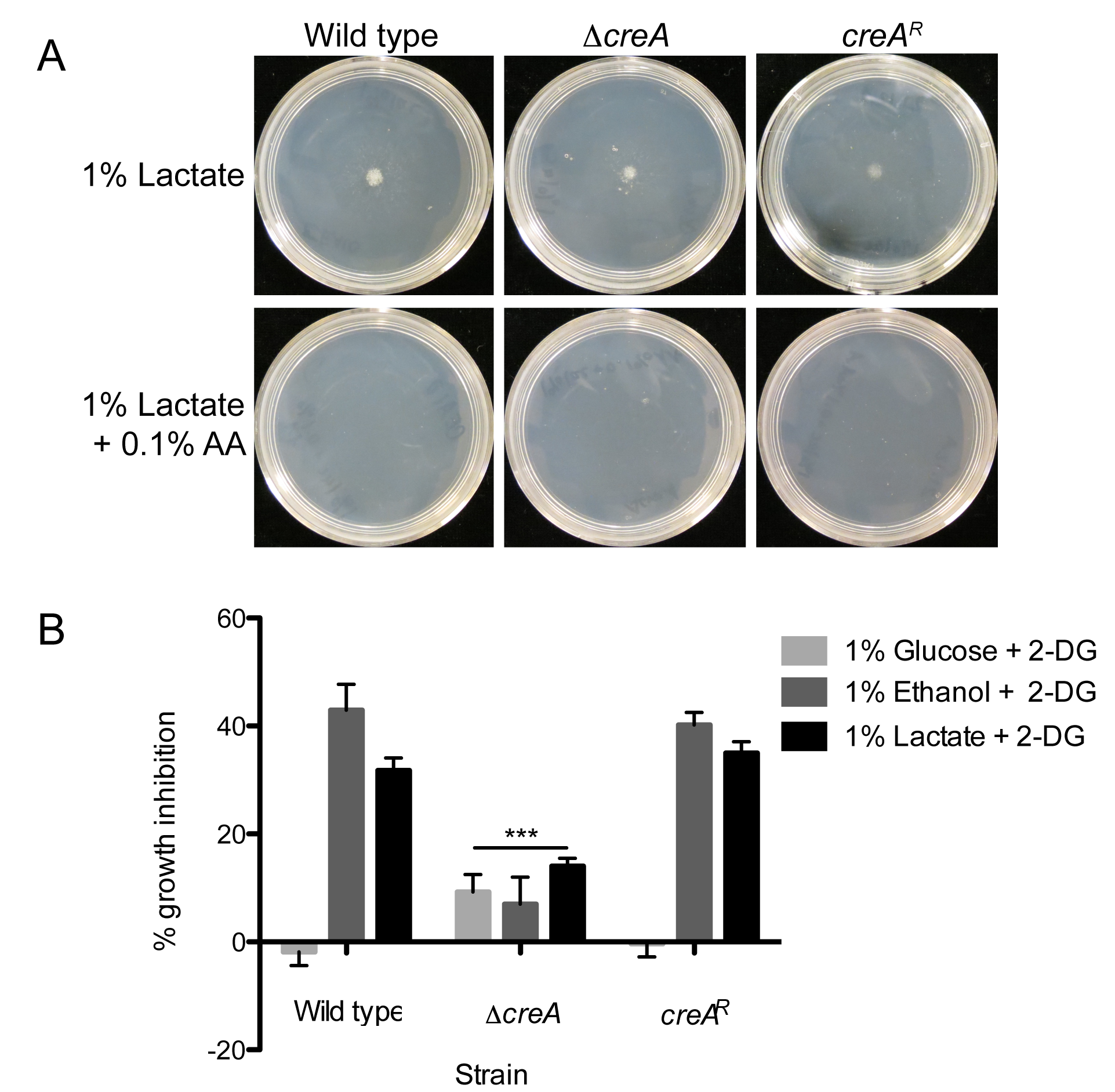

Supplement: S3 Fig — A) Growth of strains on 1% lactate minimal media with or without 0.1% allyl alcohol (AA) and incubated for 48 hours. b) Growth inhibition of CEA10, ΔcreA and creAR on indicated carbon source with 0.1% 2-deoxyglucose (2-DG) for 72 hours. Data represents mean of biological triplicates ± SEM; ***p<0.0001 by unpaired, two-tailed t-test as compared to WT of respective condition. creAR strains used for this experiment is creAR A. All assays use 1x10^3^ spore dilutions incubated at 37°C. (TIF) [file ppat.1006340.s003.tif]

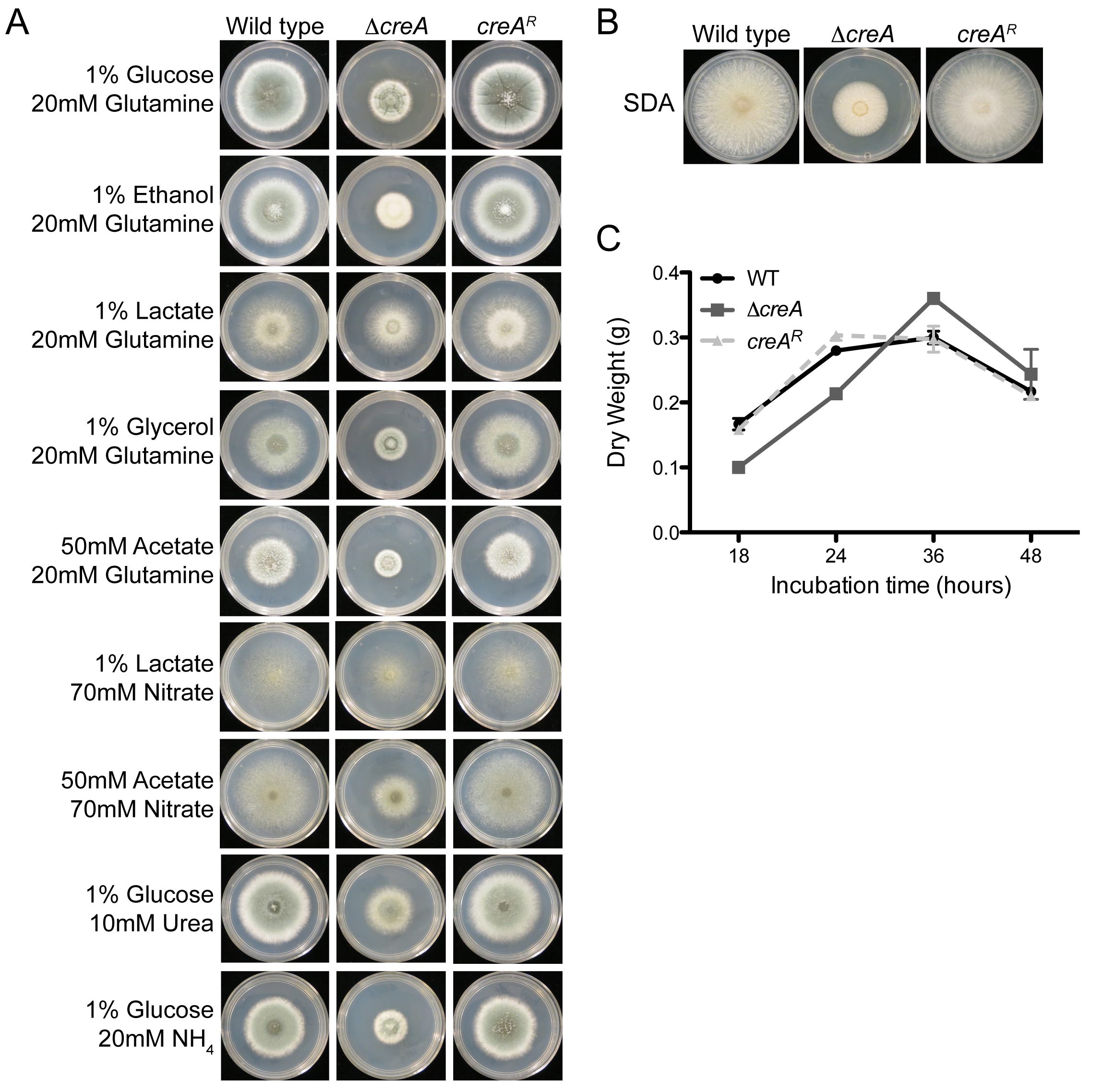

Supplement: S4 Fig — A) Growth of 1x10^3^ WT, ΔcreA and creAR conidia on media containing the indicated carbon and nitrogen sources or (B) Sabouraud Dextrose agar (SDA) for 72 hours at 37°C. C) Biomass (as measured by dry weight) of WT, ΔcreA and creAR over 48 hours from cultures of 5x10^5^ conidia/mL, grown at 37°C with shaking at 200rpm. (TIF) [file ppat.1006340.s004.tif]

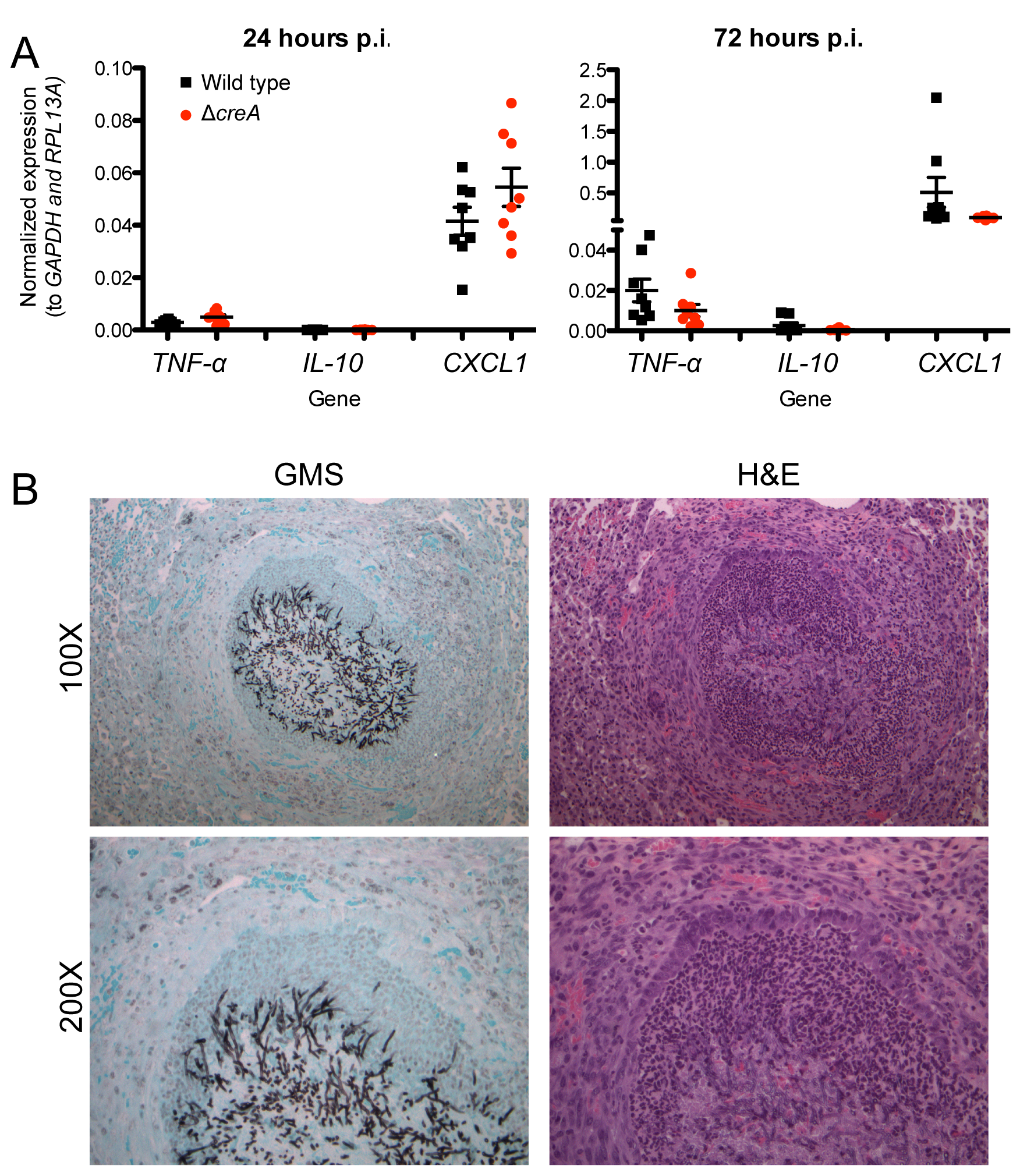

Supplement: S5 Fig — A) Expression of cytokine genes from triamcinolone treated mice, collected 24 or 72 hpi with 5x10^7^ wild type or ΔcreA conidia. Data represents eight biological replicates ± SEM. Gene expression is normalized to GAPDH and RPL13A. B) GMS and H&E staining of histological sections of lung tissue from ΔcreA survivors of triamcinolone model of IPA (see Fig 2A for experimental details), collected 14 days post inoculation. (TIF) [file ppat.1006340.s005.tif]

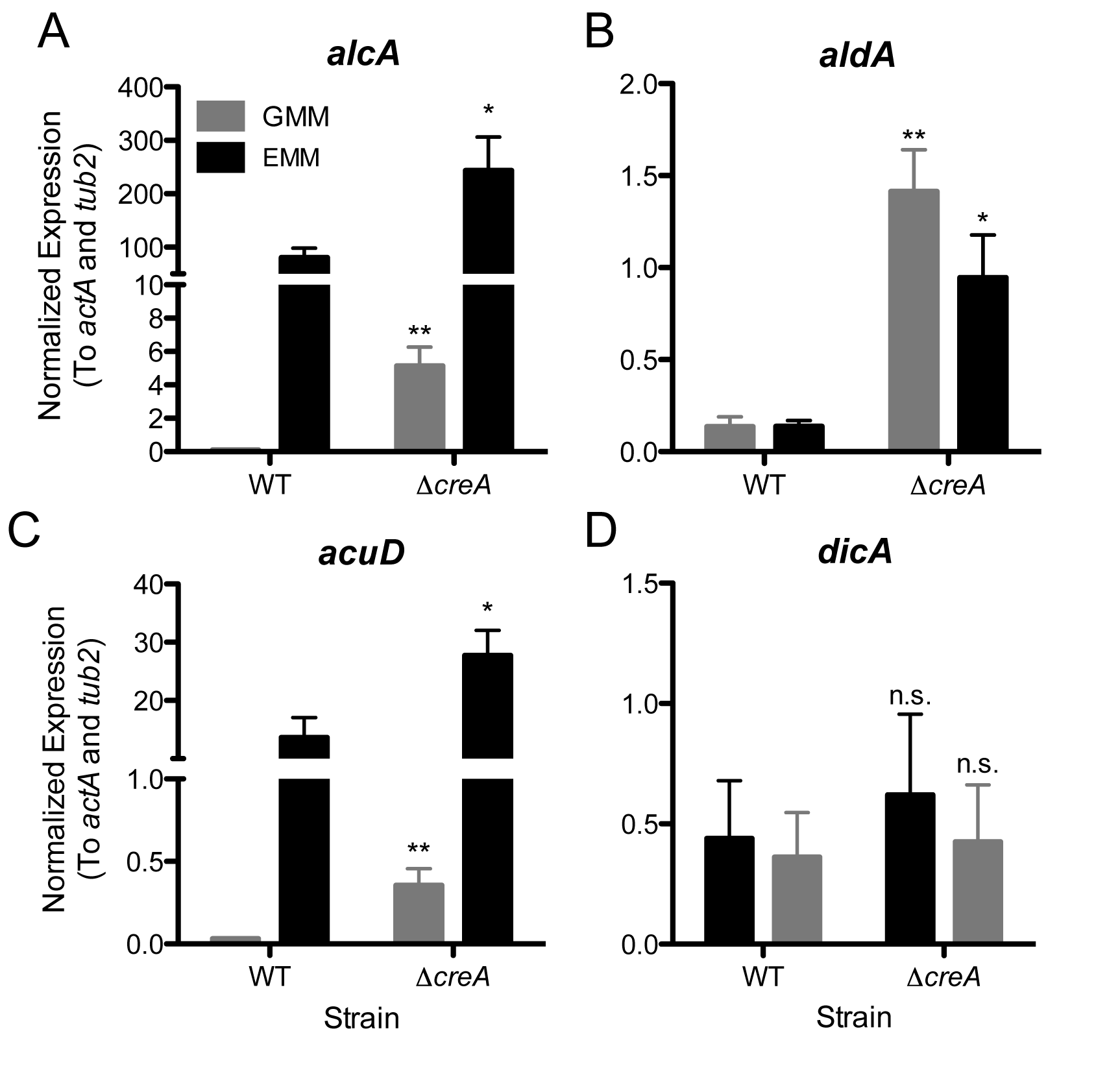

Supplement: S6 Fig — Expression analysis of A) alcA, B) aldA, C) acuD, which were significantly increased in ΔcreA compared to wild type in our RNA-sequencing analysis. D) Expression of dicA, which did not significantly change in ΔcreA in the RNA-sequencing analysis. Expression of each gene is normalized to tub2 and actA from cultures grown overnight in 1% glucose, then shifted to 1% glucose (GMM) or 1% ethanol (EMM) minimal media for 2 hours. Error bars represent SEM across four biological replicates. A) **p = 0.0044, *p = 0.0465; B) **p = 0.0015, p = 0.0134; C) *p = 0.0189, **p = 0.0423; D) n.s. = not significant by unpaired, two-tailed t-test as compared to WT of respective conditions. (TIF) [file ppat.1006340.s006.tif]

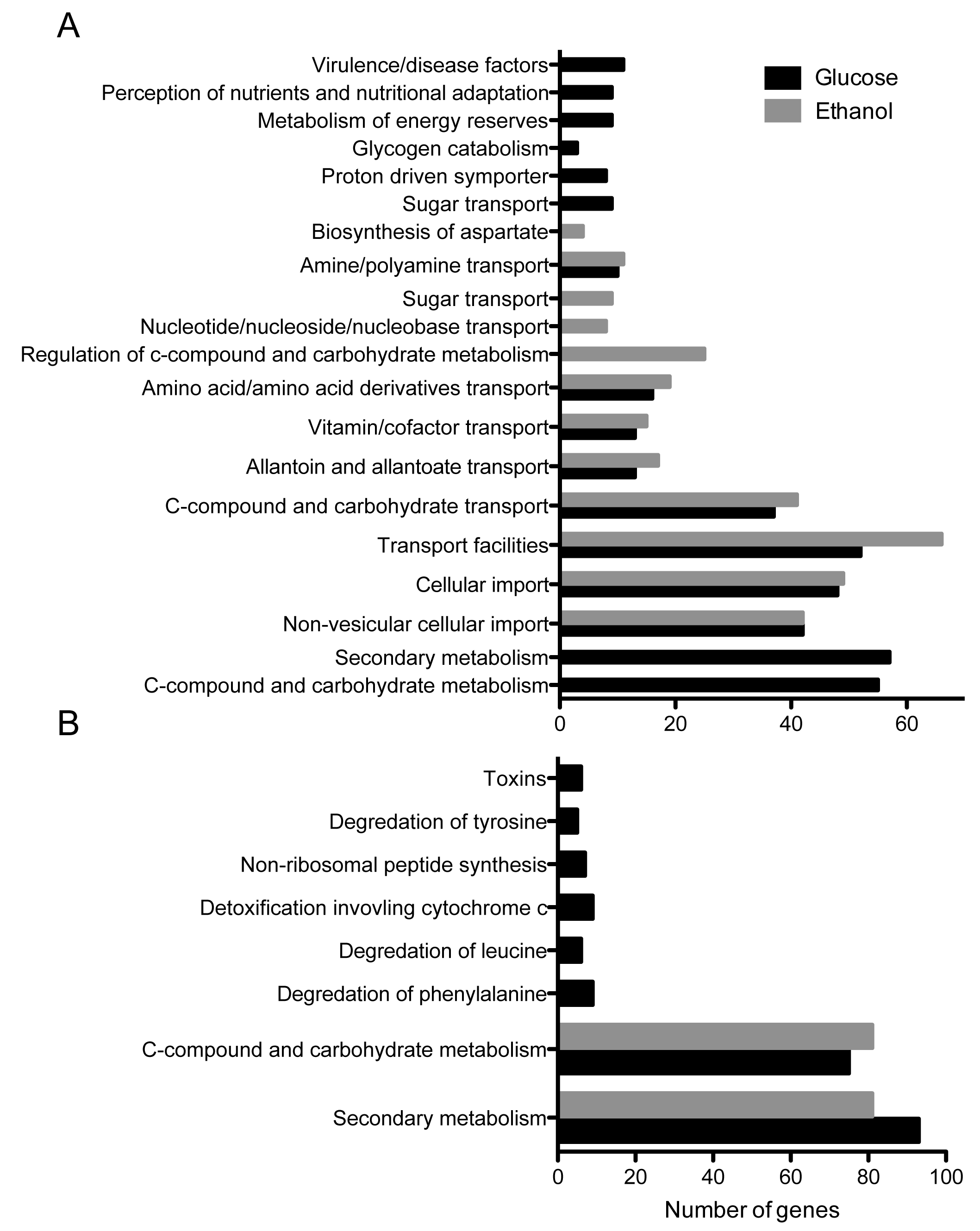

Supplement: S7 Fig — FunCat category enrichment of genes that significantly increase (A) or decrease (B) by at least 2-fold (p<0.05) in ΔcreA versus WT for 1% glucose and 1% ethanol conditions. Generated using FungiFun2 [26]. (TIF) [file ppat.1006340.s007.tif]

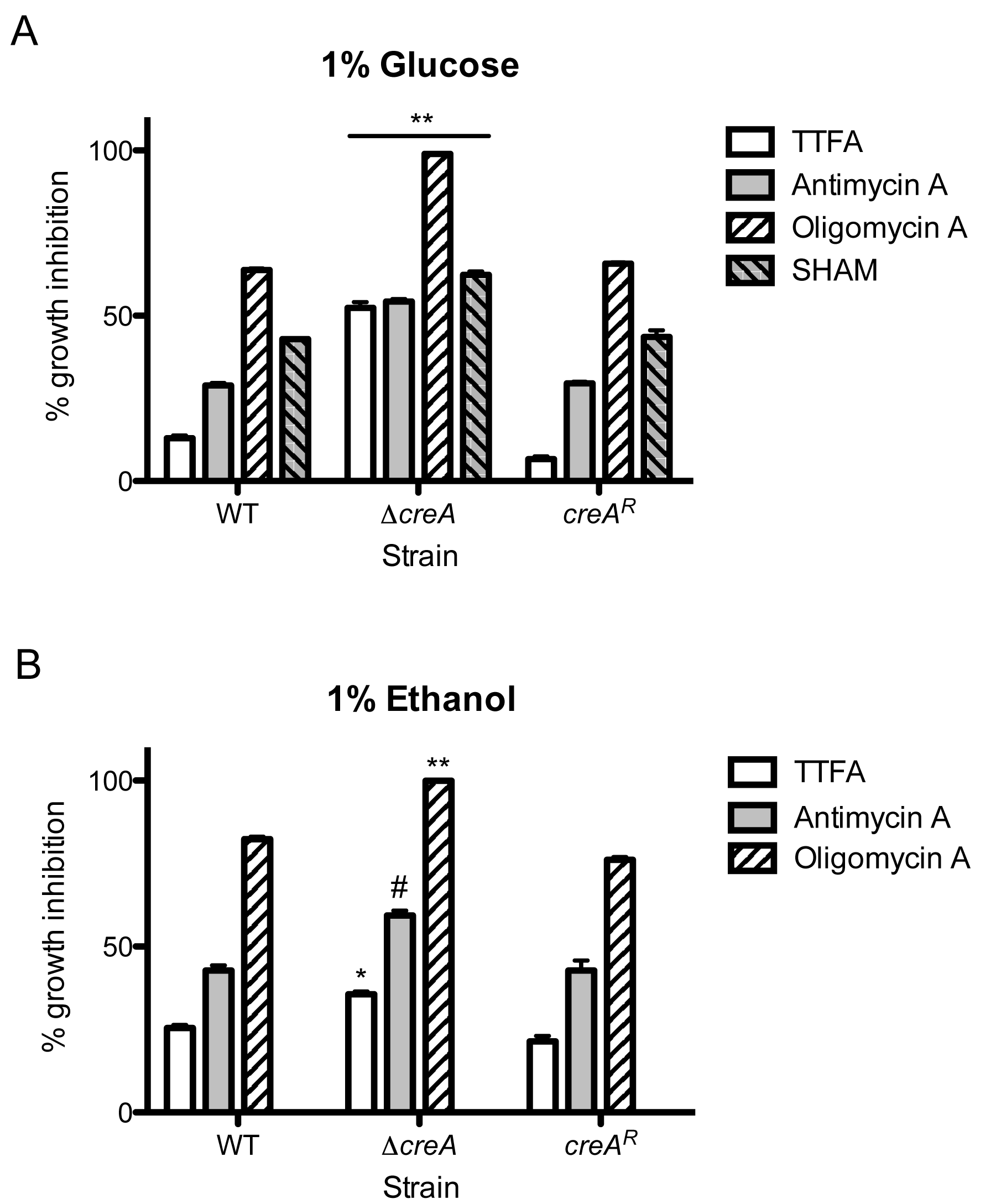

Supplement: S8 Fig — Percent growth inhibition of WT, ΔcreA and creAR grown on (A) GMM or (B) EMM in the presence of 0.1mM thenoyltrifluoroacetone (TTFA), 15ug/mL Antimycin A, 10uM Oligomycin A, or 5mM Salicylhydroxamic acid (SHAM) for 72 hours at 37°C. Data represents mean of biological triplicates ± SEM; **p<0.0001; *p = 0.0012; #p = 0.0015 by unpaired, two-tailed t-test as compared to WT of respective conditions. creAR strain used for this experiment is creAR A. (TIF) [file ppat.1006340.s008.tif]
